# Decoding thermal adaptation: genome-wide identification of TRP channel repertoire in cephalopods and gene expression responses to chronic thermal exposure in *Sepia officinalis* and *Octopus maya* embryos

**DOI:** 10.3389/fphys.2026.1851308

**Published:** 2026-07-13

**Authors:** Luis M. Molina-Carrillo, Marina Morini, Sylvie Dufour, Pavel Galindo-Torres, Sadot Ramos-Rodriguez, Carlos Rosas, Claudia Caamal-Monsreal, Clara E. Galindo-Sánchez, Gaëtan Schires, Yann Bassaglia, Laure Bonnaud-Ponticelli

**Affiliations:** 1 Laboratoire de Biologie des Organismes et des Écosystèmes Aquatiques-BOREA, Muséum national d’Histoire naturelle (MNHN), SU, CNRS, IRD, UA, Paris, France; 2 Centro de Investigación Científica y de Educación Superior de Ensenada (CICESE), Ensenada, Baja California, Mexico; 3 Unidad Multidisciplinaria de Docencia e Investigación (UMDI), Facultad de Ciencias, Universidad Nacional Autonóma de México (UNAM), Hunucma, Yucatán, Mexico; 4 Station Biologique de Roscoff, CNRS-Sorbonne Université, Roscoff, France; 5 Université Paris Est Créteil-Val de Marne (UPEC), Paris, France

**Keywords:** cephalopods, expression profiles, molecular phylogeny, thermal stress, TRP channels

## Abstract

Transient Receptor Potential (TRP) channels constitute a versatile family of membrane proteins central to sensory perception and a broad range of physiological functions. Conserved throughout evolution in both vertebrates and invertebrates, TRPs respond to diverse environmental stimuli, including temperature fluctuations, mechanical forces, osmotic changes, redox states, and chemical signals. In cephalopods, marine invertebrates renowned for their sophisticated sensory systems and behavioural complexity, the molecular mechanisms underlying thermal sensing remain poorly investigated. In this study, we present the first genome-wide identification and phylogenetic classification of 157 candidates TRP channel sequences among them 117 previously unannotated from 13 cephalopod species. These sequences were phylogenetically assigned to seven major TRP channel families (TRPA, TRPN, TRPC, TRPM, TRPV, TRPML, and TRPP). We further analysed TRP gene expression under chronic thermal stress in embryos of *Sepia officinalis* and *Octopus maya*, two species of ecological and economical relevance and occupying contrasting thermal niches. Our findings reveal a remarkably diverse TRP repertoire in cephalopods and identify αTRPC as the most consistently and robustly upregulated subtype in response to elevated temperature in both *S. officinalis* and *O. maya*. Strikingly, αTRPC expression is markedly enriched in the eyes of both species, suggesting a potential integrative function in processing both thermal and visual cues, an adaptive feature likely beneficial in rapidly changing coastal environments. This work provides the first in-depth characterisation of TRP channels in cephalopods and advances our understanding of the molecular basis of thermal adaptation in marine invertebrates. These results lay a foundation for future functional investigations and contribute to a broader understanding of how cephalopod sensory systems may respond to ongoing ocean warming.

## 1 Introduction

Global warming, primarily caused by anthropogenic activities, has increased global temperatures, with the world’s oceans and coastal regions undergoing particularly significant changes. As major heat sinks, oceans have absorbed over 90% of the excess heat generated by greenhouse gas emissions, resulting in persistent increases in sea surface temperatures (IPCC, 2023). In 2025, for instance, the average temperature of the ocean surface was recorded at 1.03 ± 0.05 °C above the 1850–1900 preindustrial baseline (Berkeley Earth, 2026). This sustained warming is already disrupting physical and biological oceanic processes, reshaping marine and coastal ecosystems, and posing a serious threat to global biodiversity (Hoegh-Guldberg et al., 2018). These changes are affecting marine ecosystems at multiple biological and ecological levels, including species distributions, community composition, trophic interactions, and ecosystem functioning (Bozinovic and Pörtner, 2015; IPCC, 2021, 2023; Pörtner, 2010).

Coastal zones, which serve as dynamic interfaces between terrestrial and marine environments, are particularly vulnerable to intensified thermal stress. These areas are critical habitats for numerous aquatic ectotherms that rely on shallow coastal waters for reproduction. Many of these organisms undergo early developmental stages, such as embryogenesis, that are characterised by low or absent mobility, rendering them especially susceptible to environmental fluctuations (Przeslawski, 2004; Khan et al., 2020). Among these, cephalopod eggs, in which embryogenesis progresses, are considered one of the most vulnerable life stages due to their sessile nature and prolonged exposure to variable and often extreme thermal conditions (Boletzky, 1986; Melzner et al., 2009).

Despite the high thermal sensitivity observed during embryonic and early developmental stages, many adult cephalopod species exhibit considerable physiological plasticity and relatively broad thermal tolerance ranges. In several marine regions worldwide, rising sea temperatures have been associated with distributional shifts, increased population abundance, and ecological niche expansion across diverse cephalopod taxa, including squids, cuttlefish, and octopuses (Doubleday et al., 2016). This adaptive capacity has been partly linked to their exceptional molecular plasticity, including extensive post-transcriptional regulation and RNA editing mechanisms that may contribute to rapid adaptation to changing environmental conditions, including thermal variability (Liscovitch-Brauer et al., 2017). Together, these observations suggest that cephalopod thermal biology results from a complex balance between vulnerability during early developmental stages and a strong adaptive potential in later life stages.

In this context, cephalopods provide a compelling model for exploring physiological and behavioural adaptations to dynamic coastal environments. Their reproductive strategy, characterised by laying eggs in shallow coastal or intertidal zones, subjects developing embryos to pronounced fluctuations in abiotic factors, such as temperature, salinity, and light exposure. To successfully navigate these challenges, cephalopods possess a highly developed and diversified sensory repertoire that enables them to detect and respond to a wide range of environmental cues. This sensory system comprises specialised receptors and organs distributed throughout the body and includes: (1) photoreception, mediated through both ocular and extraocular pathways involving light-sensitive molecules such as opsins (Kingston et al., 2015; Bonadè et al., 2020); (2) chemoreception, occurring through chemotactile receptors related to acetylcholine receptor families in arm suckers and ionotropic receptors-based olfactory pathways in olfactory organs (Budelmann, 1996; Van Giesen et al., 2020; Andouche et al., 2021; Allard et al., 2023), (3) mechanoreception, facilitating the detection of pressure changes, vibrations, and mechanical displacement through mechanosensory organs like the skin, suckers, and statocysts (York et al., 2016). In addition to these sensory modalities, increasing evidence suggests that cephalopods possess sophisticated mechanisms for detecting and responding to potentially harmful stimuli. Experimental studies have demonstrated long-lasting behavioural changes, wound-directed responses, and sensitization following tissue injury, supporting the existence of complex nociceptive pathways in these animals (Crook and Walters, 2011; Crook, 2022). These findings further highlight the remarkable sensory capabilities of cephalopods and raise important questions regarding the molecular mechanisms underlying the detection of environmental stressors, including temperature. Despite these advances, the mechanisms underlying thermoreception in cephalopods remain poorly understood.

Temperature represents a pervasive environmental stressor with direct consequences for reproductive success, affecting gametogenesis, spawning, embryonic development, and hatchling viability in several cephalopod species (Domingues et al., 2002; Rodhouse et al., 2014, 2014; Juárez et al., 2015; Caamal-Monsreal et al., 2016; O’Brien et al., 2017). Understanding how cephalopods cope with thermal variations is therefore essential for assessing their vulnerability and resilience under climate change scenarios. While the induction of heat shock proteins (HSPs) is one of the best-characterised molecular responses to heat stress in cephalopods (Hong et al., 2015; Long et al., 2015), the primary mechanisms by which thermal changes are detected and transduced into cellular responses remain largely unexplored.

The detection of thermal variation is mediated by thermoreceptors, including a set of specialised ion channels that respond to specific temperature ranges (Chiang et al., 2023). Among these, the transient receptor potential (TRP) channels constitute a diverse superfamily, which is a group of multifunctional cell membrane proteins characterised by their six transmembrane segments modulating intracellular Ca^2+^ concentrations (Himmel et al., 2020; Himmel and Cox, 2020). The TRP superfamily members have been generally classified into nine TRP families in metazoans based on the homology of their amino acid sequences: TRPA (TRP ankyrin), TRPC (TRP canonical), TRPM (TRP melastatin), TRPML (TRP mucolipin), TRPN (TRP nompC, or no mechanoreceptor potential C), TRPP (TRP polycystin or polycystic kidney disease), TRPS (TRP soromelastatin), TRPV (TRP vanilloid), and TRPVL (TRP vanilloid-like) (Chiang et al., 2023; Himmel and Cox, 2020; Zhang, 2015).

The functional significance of TRP channels in thermosensation was recognised with the awarding of the 2021 Nobel Prize in Physiology or Medicine to David Julius and Ardem Patapoutian, for the discovery of receptors involved in temperature and mechanosensation. In particular, TRPV1 was identified as a key detector of noxious heat, whereas TRPM8 plays a central role in cold sensation. TRP genes are widely present across the eukaryotic kingdom, extending beyond all principal vertebrate lineages, such as fish, amphibians, reptiles, birds, and mammals, to encompass diverse non-vertebrate phyla, including arthropods (e.g., insects and crustaceans), nematodes, and molluscs (Montell and Rubin, 1989; Patapoutian et al., 2003; Jerônimo et al., 2017; Fu et al., 2021; Takahashi et al., 2021; Zhang et al., 2023). TRPs were shown to be activated by diverse stimuli, including osmotic pressure, chemical signals, mechanical forces, and temperature variations, and to mediate various physiological responses such as regulation of inflammation, cardiovascular function, calcium homeostasis, lysosomal activity, cell proliferation, and apoptosis (Schwarz et al., 2019; Himmel and Cox, 2020; Fu et al., 2021; Jiang et al., 2023).

Recently, genome-wide studies in bivalve molluscs have identified multiple members of the TRP channels superfamily and examined their expression profiling under heat stress conditions. In particular, expression analyses in *Chlamys farreri*, *Ruditapes philippinarum, and Magallana gigas* (formerly *Crassostrea gigas*), across several tissues (including mantle, gill, foot and adductor muscle, and additionally the eyes in *C. farreri*), indicate that members of the TRPA, TRPC, and TRPV families are associated with molecular response to heat stress, while some TRPM genes are mainly associated with optimal and cold temperature conditions (Fu et al., 2021; Peng et al., 2021; Zhang et al., 2023). However, in cephalopods, despite the availability of several genome assemblies and annotated TRP channel sequences in public databases (e.g., NCBI, UniProt), broad-scale surveys of TRP channel diversity and systematic expression analysis under thermal stress conditions remain largely unexplored.

In this study, we present comprehensive genome-wide identification and phylogenetic characterisation of the Transient Receptor Potential (TRP) channel repertoire across 13 cephalopod species, providing a foundational framework for future comparative analyses. We examine the expression profiles of various TRP genes in embryos of two ecologically and economically important cephalopod species: *Sepia officinalis*, from the temperate waters of the Northeast Atlantic (notably the English Channel), and *Octopus maya*, endemic to the warm waters of the Gulf of Mexico. Gene expression was analysed under chronic exposure to both optimal (16 °C for *S. officinalis* and 24 °C for *O. maya*) and heat stress (22 °C and 30 °C, respectively) temperature regimes. TRP transcripts were quantified in whole embryos as well as across four tissues (eyes, skin, brain, and optic lobes) allowing for a spatially resolved analysis of thermal sensitivity. By comparing the expression profiles of heat-responsive TRP transcripts between these two thermally distinct species, we aimed to identify candidate TRP channels potentially involved in mediating adaptive responses to thermal stress. This study offers new perspectives on TRP channels potentially mediating thermal sensitivity in marine cephalopods, while shedding light on the mechanisms that may support their adaptive resilience in the face of ongoing and future climate change.

## 2 Materials and methods

### 2.1 Genome-wide identification of TRP genes in cephalopods

To identify the complete set of TRP genes in cephalopods, we first compiled a reference dataset of already annotated TRP protein sequences from the NCBI and UniProt databases. This dataset included representatives from non-vertebrate taxa, focusing particularly on four cephalopod species (*Octopus bimaculoides*, *O. vulgaris*, *O. sinensis*, and *Acanthosepion pharaonis*), as well as other molluscan lineages, namely the gastropod *Haliotis rufescens* and the bivalves *Crassostrea virginica*, *Magallana gigas*, and *Pecten maximus*. To broaden the phylogenetic framework within Lophotrochozoa, TRP sequences from the annelids *Galeolaria caespitosa* and *Lamellibrachia satsuma* were also included. Additional representatives from Ecdysozoa comprised the arthropods *Drosophila melanogaster*, *Daphnia pulex*, and *Daphnia magna*, as well as the nematode *Caenorhabditis elegans*. Finally, to provide a vertebrate perspective, we incorporated sequences from cyclostomes (*Lampetra planeri*, *Lampetra fluviatilis*, *Lethenteron reissneri*, and *Myxine glutinosa*) and gnathostomes, represented by *Lepisosteus oculatus* and *Homo sapiens*.

To identify TRP genes in cephalopods, we analyzed the predicted proteomes derived from 13 cephalopod genomes available in the NCBI genome database. These included species from the orders Octopoda (e.g., *Octopus maya:* GCA_027122535.1*, Octopus vulgaris:* GCA_951406725.2*, Octopus sinensis:* GCA_006345805.1*, Octopus bimaculoides:* GCA_001194135.2), Sepiida (e.g., *Sepia officinalis:* GCA_964300435.1, *Acanthosepion pharaonis* (*Sepia pharaonis:* GCA_903632075.3)*, Acanthosepion esculentum* (*Sepia esculenta:* GCA_964036315.1)*, Ascarosepion bandense* (*Sepia bandensis:* GCA_037127315.1)), Teuthida (suborder Oegopsida, e.g., *Architeuthis dux:* GCA_006491835.1; suborder Myopsida, e.g., *Doryteuthis pealeii:* GCA_023376005.1*, Doryteuthis opalescens:* GCA_043791155.1), Sepiolida (e.g., *Euprymna scolopes:* GCA_024364805.1), and Nautilida (e.g., *Nautilus macromphalus*: GCA_976954165.2). The curated reference TRP sequences described above were used as queries in local BLASTP searches against each cephalopod proteome, using an E-value threshold of 1 × 10^−5^. Candidate TRP proteins retrieved from these searches were manually inspected by evaluating sequence length, domain organization, and transmembrane architecture, and by comparison with homologous TRP proteins from closely related species. Candidate sequences were subsequently validated through conserved domain analyses using the Conserved Domain Database (CDD) (www.ncbi.nlm.nih.gov/Structure/cdd/wrpsb.cgi), InterPro (www.ebi.ac.uk/interpro), and the HMMER program (www.ebi.ac.uk/Tools/hmmer/). Sequences lacking characteristic TRP domains or corresponding to incomplete or erroneous gene predictions were excluded from subsequent analyses. When multiple highly similar sequences or alternative isoforms were associated with the same genomic locus, a representative sequence was selected for downstream phylogenetic analyses. This approach was adopted to minimise redundancy and avoid overrepresentation of individual loci within the phylogenetic dataset. Representative sequences therefore corresponded to unique, manually validated loci, whereas redundant isoforms and incomplete predictions were not retained in the final dataset. The molecular weight (MW) and theoretical isoelectric point (pI) of each validated protein were calculated using ProtParam, and subcellular localisation was predicted using Cell-PLoc 2.0. Detailed information on the validated TRP repertoire is provided in **Supplementary Table 1**, and conserved-domain architectures are shown in **Supplementary Figures 5–10**.

### 2.2 Phylogenetic analysis and classification of TRP genes

Phylogenetic analysis was performed using all TRP protein sequences identified in the 13 cephalopod species analyzed in this study, together with a reference dataset of previously annotated TRP proteins representing major metazoan lineages. The reference dataset included representatives from Mollusca, Annelida, Arthropoda, Nematoda, Cyclostomata, and Gnathostomata, providing a evolutionary framework for the classification of cephalopod TRP proteins. Rather than reconstructing the complete metazoan TRP repertoire, the objective of the phylogenetic analysis was to classify cephalopod TRP sequences within the major recognized TRP channel families using well-characterized homologues from representative taxa.

Multiple sequence alignments were generated using Clustal Omega (Sievers et al., 2011) implemented in SeaView 5.0.1. Phylogenetic relationships were inferred using both Maximum Likelihood (ML) and Bayesian Inference (BI) approaches. ML analyses were performed using RAxML (Stamatakis, 2014) under the JTT amino acid substitution model with 1,000 bootstrap replicates to assess node support. Bayesian phylogenetic reconstruction was conducted using MrBayes v3.2.7a (Ronquist et al., 2012) through the CIPRES Science Gateway. Two independent Markov chain Monte Carlo (MCMC) runs were performed using four chains (one cold and three heated chains) for 10 million generations, with trees sampled every 10,000 generations. The first 25% of sampled trees were discarded as burn-in, and posterior probabilities were estimated from the remaining trees. The resulting phylogenetic trees were rooted using the Piezo sequence from *Drosophila melanogaster* (NM_001273298.1) as the outgroup and visualized using iTOL (Letunic and Bork, 2021). Node support was evaluated using bootstrap percentages from the ML analyses and posterior probabilities from the Bayesian analyses. Accession numbers for all TRP proteins included in the phylogenetic analyses are provided in **Supplementary Table 1** and **Supplementary Figure 4**.

### 2.3 Embryo collection and experimental design

#### 2.3.1 Sepia officinalis

Three distinct batches of cuttlefish eggs were collected from the Channel Sea Coast in Roscoff, France, in May 2024. All specimens were transported to Roscoff marine station (CRB-Sorbonne Université, EMBRC-France) and kept in an open circulatory system with filtered seawater at a temperature of 16.3 ± 0.6 °C under a 12-hour light/12-hour dark photoperiod conditions, salinity of 35.6 ± 1.4 parts per thousand (ppt), and pH of 8.1 ± 0.06. Ammonia, nitrite, and nitrate levels were monitored weekly using colourimetric tests (Macherey-Nagel™).

The embryonic development of *S. officinalis* is divided into 30 distinct stages (Boletzky et al., 2016). Experimental treatments commenced at developmental stages 23–24 (just before the beginning of the eye pigmentation and when photosensitive molecular pathways come into play (Bonadè et al., 2020)) using a total of 360 embryos (120 eggs per spawn from three distinct spawns), which were randomly assigned to two thermal conditions: 16 ± 0.4 °C, considered optimal, and 22 ± 0.6 °C, identified as a thermally stressful condition (Bouchaud, 1991; Melzner et al., 2006, 2007; Bloor et al., 2013). Thermal stress was induced by gradually increasing the incubation temperature by 2 °C per day until the target temperature of 22 °C was reached, after which it was maintained consistently for the duration of the experiment.

Embryos were extracted from the egg capsule in filtered seawater and staged according to standard criteria established by Boletzky et al. (2016). To evaluate the effects of elevated temperature on TRP transcript levels, *Sepia officinalis* embryos were maintained at either 16 °C or 22 °C and sampled at developmental stage 30 immediately prior to hatching. Sampling was performed after approximately 44 days at 16 °C and 20 days at 22 °C, corresponding to the time required for embryos to reach stage 30 under each temperature condition. Immediately after staging, samples were preserved in RNAlater (Life Technologies, Carlsbad, CA, USA) for subsequent gene expression analyses and stored at −80 °C until RNA extraction.

#### 2.3.2 Octopus maya

Wild mature male and female octopuses were captured from the continental shelf of the Yucatán Peninsula. All specimens were transported, maintained, and acclimated following the procedures described by Galindo-Torres et al. (2024).

The embryonic development of *O. maya* is divided into 20 distinct stages (Naef, 1928; Castro-Fuentes et al., 2002). Experimental treatments began at developmental stages 5-6, using a total of 240 eggs (from three different spawns), which were randomly assigned to two incubation temperatures: 24 ± 0.8 °C, considered optimal, and 30 ± 1.1 °C, identified as a thermally stressful condition (Juárez et al., 2015; Caamal-Monsreal et al., 2016). Thermal stress was induced by gradually increasing the incubation temperature by 1 °C per day until reaching 30 °C, after which it was maintained consistently for the duration of the experiment.

To assess the impact of elevated temperature on TRP transcript levels, embryos were sampled at stage 19, immediately prior to hatching. Sampling was performed after approximately 30 days of exposure at 30 °C and 45 days at 24 °C, corresponding to the time required for embryos to reach the selected developmental stage under each temperature condition. Following removal from the egg capsule in filtered seawater, embryos were staged according to established morphological criteria (Naef, 1928; Castro-Fuentes et al., 2002). Prior to RNA extraction, embryos were visually examined and only individuals without externally detectable morphological abnormalities were retained for subsequent gene expression analyses. Developmental abnormalities previously reported in *O. maya* embryos exposed to elevated temperatures are described in detail by Caamal-Monsreal et al. (2016) and Galindo-Torres et al. (2024).

### 2.4 Extraction, DNase treatment, and reverse transcription

For each temperature condition, ten embryos of *Sepia officinalis* and *Octopus maya* were sampled for molecular analysis. Five embryos of each species were processed as whole organisms, while the remaining five were dissected to isolate specific tissues, including eyes, brain, optic lobes and dorsal skin. Total RNA from all the samples was extracted using the NucleoSpin R.N.A. midi kit (Macherey Nagel, Düren, Germany) following the manufacturer’s protocol. DNase treatment of each extract was carried out according to the Ambion Turbo DNA-free™ Kit (Ambion, Applied Biosystems, Darmstadt, Germany). Then, they were purified with NucleoSpin R.N.A. Clean-up (Macherey Nagel). Quantity and quality were assessed with Qubit 3 fluorimeter (Invitrogen). RNA integrity was confirmed by a 1.2% denaturant agarose-formaldehyde gel. Samples were subsequently stored at −80 °C until further use.

Single-strand cDNA was synthesised with SuperScript™ III First-Strand Synthesis System kit for RT-PCR (Invitrogen, Carlsbad, CA, USA), following the manufacturer’s instructions.

### 2.5 Primer design and selection

Specific primers for TRP genes were designed by combining NCBI and Primer3 software (Rozen and Skaletsky, 1999), in accordance with the QIAcuity Application Guide (www.qiagen.com/fr/resources) (**Supplementary Table 2**). They were tested using cDNA from cuttlefish eyes, skin, and central nervous system (CNS = brain and optic lobes). The PCR mix includes REDTaq, PCR Reaction Mix (Eurogentec, Seraing, Belgium), and 10 μM of each primer in a final volume of 50 μL. The thermocycler program was: 5 min at 95 °C, 40 cycles of 30 s at 95 °C, 1 min at 58 °C, 30 s at 72 °C and the final extension of 2 min at 72 °C. For the visualisation of PCR products, 2% agarose gel was used.

### 2.6 Gene expression analysis using digital PCR

A QIAcuity Digital PCR System (Qiagen, Hilden, Germany) was used to perform absolute quantification of gene expression by using the QIAcuity EG PCR Kit (Cat No. 250113; Qiagen) and 8.5K 96-well Nanoplates (Cat No. 250021; Qiagen). The QIAcuity 8.5K 96-well Nanoplates are microfluidic dPCR plates that process 96 samples with up to 8.5K partitions/well. The PCR reaction occurred in each partition, and the partition volume was 0.34 nL. The dPCR analyses were performed in a final volume of 12 μL comprising 4 μL of 3 x EG PCR Master mix buffer, 1 μL of primers (5 μM forward primer, 5 μM reverse primer), 5 μL of RNase-free water and 2 μL of template cDNA. The conditions for dPCR were as follows: 1 cycle at 96 °C for 2 min, followed by 40 cycles of 15 secs at 95 °C, 30 secs at 58 °C, and 15 secs at 72 °C, with a final cooling step for 5 min at 40 °C. Three dPCR replicates were analysed for each sample. Data were analysed using the QIAcuity Suite Software v.2.2.5 (Qiagen, Germany), and quantities were exported as Copies/μL of reaction. The dPCR assays were performed using automatic settings for threshold and baseline. The MIQE (for Minimum Information for publication of Quantitative digital PCR Experiments) guidelines are provided in **Supplementary data** (**Supplementary Table 3**).

### 2.7 Statistical analysis

Absolute quantification of TRP target transcript levels was obtained using the QIAcuity Software Suite v2.2.5 and is reported as copies/μL. For each gene, pairwise comparisons between temperatures (T1 vs T2) were evaluated using an exact two-sided permutation test, in which all possible reallocations of group labels were exhaustively enumerated and the difference in group means was used as the test statistic. To account for multiple testing across genes, p-values were adjusted using the Benjamini–Hochberg procedure to control the false discovery rate (FDR = 5%). Results are reported as mean ± SEM, and statistical significance was defined as q < 0.05. Final interpretation jointly considers statistical significance and effect size (FC = fold-change).

## 3 Results

To our knowledge, this study provides the first genome-wide identification and phylogenetic characterisation of the Transient Receptor Potential (TRP) channel repertoire across 13 cephalopod species, including numerous TRP sequences for which no prior TRP annotation was available. We further investigated TRP gene expression under experimental heat stress in *Sepia officinalis* and *Octopus maya*.

### 3.1 *In silico* identification and phylogenetic analysis of the TRP sequences

#### 3.1.1 Global phylogeny

A broad phylogenetic analysis of metazoan TRP channels revealed previously uncharacterised TRP channel types in cephalopods. This analysis encompassed 334 TRP amino acid sequences, including 250 from non-vertebrate taxa, among them multiple cephalopod species, and 84 from vertebrate models. The piezo sequence from *D. melanogaster* was used as an outgroup to root the tree.

**Figure 1:**
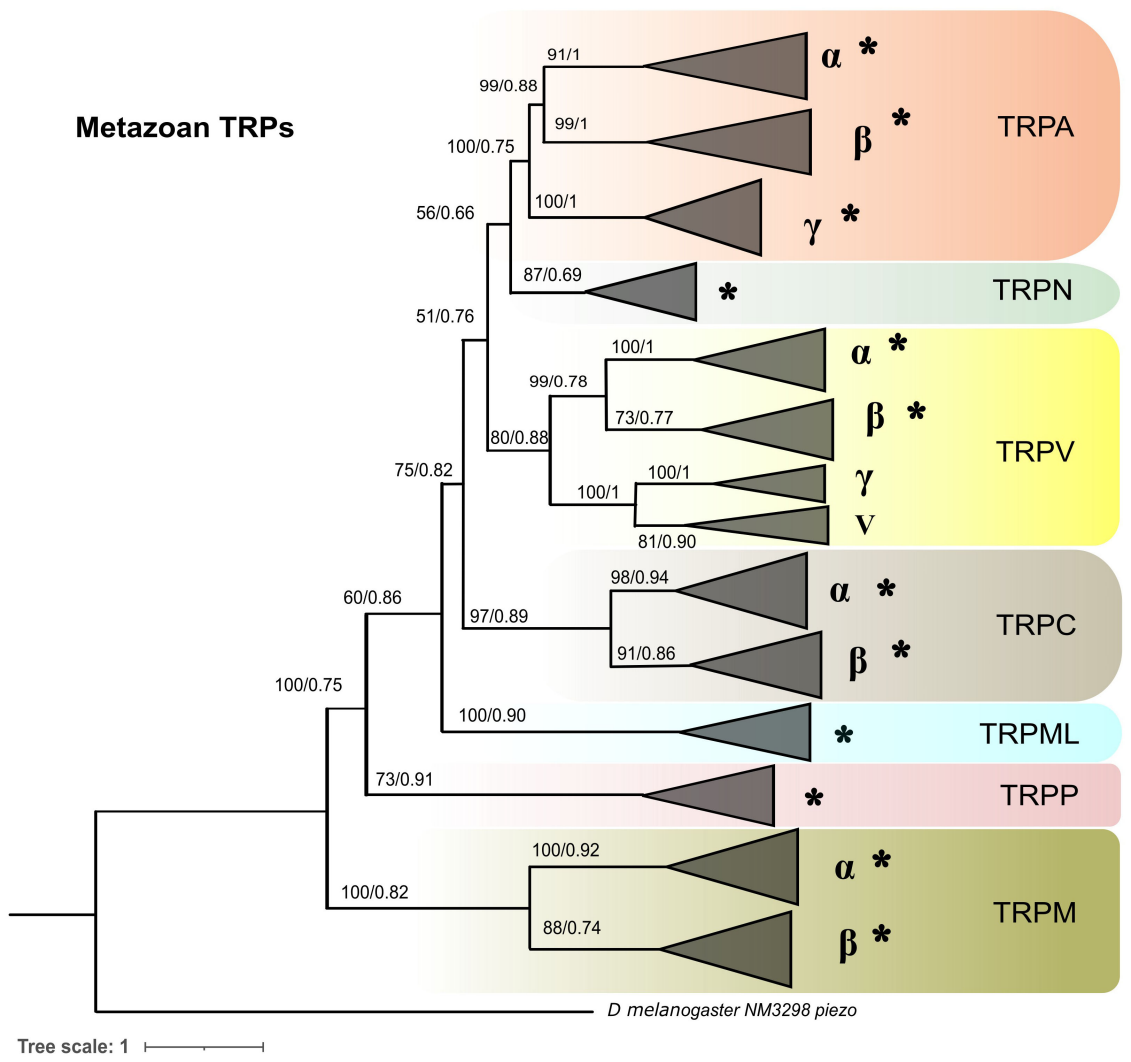
Global phylogenetic relationships of metazoan TRP channel families. The tree summarizes the relationships among representative metazoan TRP channel sequences inferred from Maximum Likelihood (RAxML) and Bayesian Inference (MrBayes) analyses. Node values correspond to Maximum Likelihood bootstrap support (%) and Bayesian posterior probabilities (BS/PP). Major TRP channel families (TRPA, TRPN, TRPV, TRPC, TRPML, TRPP, and TRPM) are colour-coded and labelled. Distinct phylogenetic groups recovered within each family are denoted by Greek letters. Asterisks (*) indicate groups containing cephalopod sequences. This global phylogeny served as the basis for the family-specific analyses presented in **Figures 2**–**8**.

The global phylogeny (**Figure 1**; **Supplementary Figure 4**), inferred using both Maximum Likelihood (RAxML) and Bayesian Inference (MrBayes) approaches, enabled the phylogenetic assignment of 117 previously unannotated TRP channel sequences across 13 cephalopod species. Based on their phylogenetic placement and similarity to reference TRP sequences from other metazoans, these sequences were assigned to seven major TRP channel families: TRPA, TRPN, TRPV, TRPC, TRPML, TRPP and TRPM. Several of these families contained distinct internal clades that were recurrently recovered across both phylogenetic approaches and subsequent family-level analyses. For descriptive purposes, these clades were designated α, β and γ where applicable. These designations are intended to facilitate comparisons among cephalopod TRP homologues and should be regarded as a working phylogenetic framework and an evolutionary hypothesis based on the currently available data, rather than as formal taxonomic or functional classifications (**Figure 1**; **Supplementary Figure 4**). These family-specific phylogenetic relationships are described in detail below.

#### 3.1.2 TRPA phylogeny

**Figure 2:**
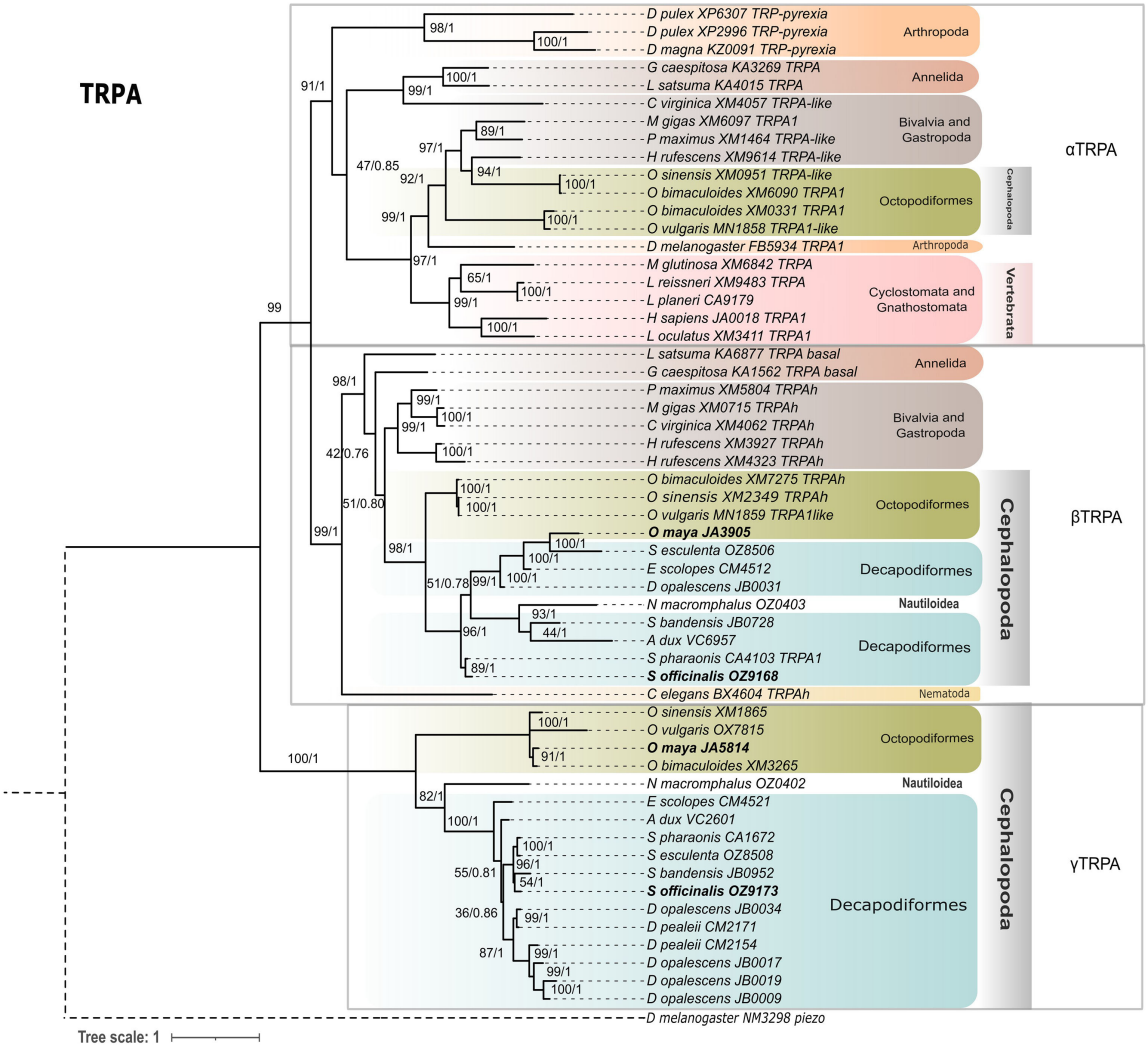
Detailed phylogeny of the TRPA channel family. The subtree recovered three principal TRPA groups, designated αTRPA, βTRPA, and γTRPA. *Sepia officinalis* and *Octopus maya* sequences are highlighted in bold. Support values are shown at the nodes.

**Figure 2** highlights the phylogenetic relationships within the TRPA (Ankyrin) channel family based on 56 amino acid sequences from a representative set of vertebrate and non-vertebrate taxa, including a broad sampling of cephalopods (**Supplementary Table 1**). Three principal TRPA clades, designated αTRPA, βTRPA, and γTRPA, which were consistently recovered by both RAxML and MrBayes. The basal nodes defining these clades were associated with high bootstrap values and posterior probabilities (αTRPA: BS = 91, PP = 1.00; βTRPA: BS = 99, PP = 1.00; γTRPA: BS = 100, PP = 1.00) (**Figure 2**).

The αTRPA clade encompasses sequences from a broad range of metazoan taxa, including arthropods, annelids, molluscs, cyclostomes, and gnathostome vertebrates. Within this clade, cephalopods αTRPA sequences were recovered exclusively from Octopodiformes, including *Octopus vulgaris*, *O. bimaculoides and O. sinensis*, where they were previously annotated as TRPA1 or TRPA1-like proteins. Outside cephalopods, TRPA1 or TRPA1-like sequences from bivalves (*Crassostrea virginica*, *Magallana gigas*, and *Pecten maximus*), gastropod *Haliotis rufescens*, annelids (*Galeolaria caespitosa* and *Lamellibrachia satsuma*), arthropods (*Drosophila melanogaster* and *Daphnia* spp.), cyclostomes (*Lampetra planeri*, *L. fluviatilis*, *Lethenteron reissneri*, and *Myxine glutinosa*), and gnathostome vertebrates represented by *Homo sapiens* and *Lepisosteus oculatus*, were grouped within clade.

The βTRPA clade, recovered as the sister group to αTRPA, comprises exclusively non-vertebrate sequences spanning a wide array of molluscan taxa (annelids, molluscs, nematodes, and cephalopods). Within cephalopods, βTRPA sequences were identified in Decapodiformes (e.g., *Sepia officinalis*, *Architeuthis dux*, *Doryteuthis opalescens*), Octopodiformes (e.g., *Octopus maya*, *O. vulgaris*) and in Nautiloidea (*Nautilus macromphalus*). The occurrence of βTRPA in *N. macromphalus* is consistent with the presence of this TRPA group prior to the divergence of Nautiloidea and Coleoidea. In addition to cephalopods, this clade includes sequences from annelids, bivalves, gastropods, and the nematode *Caenorhabditis elegans*. Notably, βTRPA includes sequences annotated as TRPA1 or TRPA1 homologues/like.

The γTRPA clade was recovered as a distinct group composed predominantly of sequences lacking prior TRPA annotation in publicly available databases. Members of this clade were identified in Decapodiformes (e.g., *Sepia officinalis*, *Architeuthis dux*, *Doryteuthis opalescens*), Octopodiformes (e.g., *Octopus maya*, *O. vulgaris*), and Nautiloidea (*Nautilus macromphalus*). The recovery of γTRPA sequences in representatives of Octopodiformes, Decapodiformes, and Nautiloidea indicates that this group is broadly distributed among the cephalopod taxa included in the present analysis. However, broader taxonomic sampling will be required to determine its distribution beyond cephalopods.

Within vertebrates, TRPA paralog diversity appears limited, represented solely within the αTRPA clade by a single TRPA1 as shown in human and spotted gar. In contrast, molluscs, and cephalopods in particular, exhibit a more diversified TRPA sequences distributed across all three clades. Notably, both βTRPA and γTRPA were recovered in representatives of Octopodiformes, Decapodiformes, and Nautiloidea, suggesting a broad distribution of these groups among extant cephalopod lineages. Furthermore, certain cephalopod species harbour duplicated paralogs within subtypes, such as for αTRPA in *O. bimaculoides* and γTRPA in *D. opalescens* and *D. pealeii*.

Based on the phylogenetic relationships inferred from both RAxML and MrBayes analyses, we propose the use of the designations αTRPA, βTRPA, and γTRPA to distinguish the three principal TRPA clades identified in the present dataset.

Detailed information on the sequences of TRPA channels and our proposed phylogenetically-based annotations is presented in **Supplementary Table 1** and **Supplementary Figure 5**.

**Figure 3:**
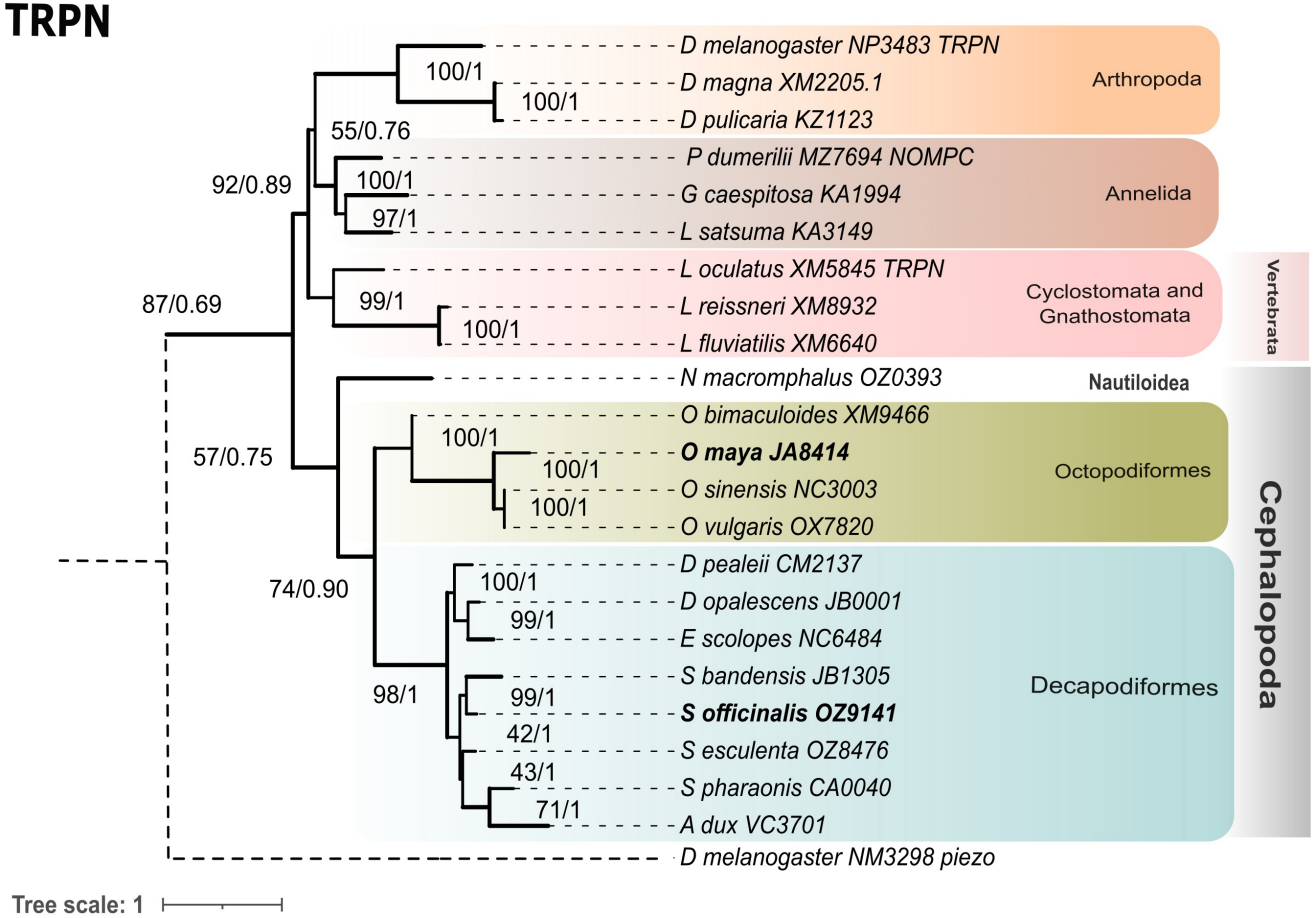
Detailed phylogeny of the TRPN channel family. The subtree identified a single principal TRPN clade. *Sepia officinalis* and *Octopus maya* sequences are highlighted in bold. Support values are shown at the nodes.

*TRPN phylogeny* **Figure 3** illustrates the phylogenetic relationships within the TRPN (No mechanoreceptor potential C or NompC) channel family based on 22 amino acid sequences from representative vertebrate and non-vertebrate taxa, including a broad sampling of cephalopods. Phylogenetic analyses recovered a single TRPN clade with moderate support (BS = 87, PP = 0.69) comprising representatives from arthropods, annelids, vertebrates, and cephalopods (**Figure 3**).

Within the cephalopod lineage, TRPN sequences were recovered from representatives of Octopodiformes (e.g., *Octopus maya*, *O. sinensis*), Decapodiformes (e.g., *Sepia officinalis*, *Doryteuthis pealeii*, *Architeuthis dux*), and Nautiloidea (*Nautilus macromphalus*). Cephalopod TRPN sequences formed a monophyletic group that was distinct from arthropod, annelid, and vertebrate homologues.

Within Cephalopoda, two principal subgroups were recovered. The first comprised octopodiform sequences, including *O. maya*, *O. vulgaris*, *O. bimaculoides*, and *O. sinensis*, which formed a highly supported clade (BS = 100, PP = 1.00). The second comprised decapodiform sequences, including representatives of Sepiida, Teuthida, and Sepiolida. *Nautilus macromphalus* occupied a position outside both octopodiform and decapodiform clades. The occurrence of TRPN homologues in representatives of Octopodiformes, Decapodiformes, and Nautiloidea indicates a broad distribution of this channel family across the cephalopod taxa included in the present analysis. Outside cephalopods, TRPN homologues were identified in arthropods (*Drosophila melanogaster*, *Daphnia magna*, and *D. pulicaria*), annelids (*Platynereis dumerilii*, *Galeolaria caespitosa*, and *Lamellibrachia satsuma*), and vertebrates represented by cyclostomes (*Lethenteron reissneri* and *Lampetra fluviatilis*) and the actinopterygian *Lepisosteus oculatus*. The recovery of TRPN sequences across these phylogenetically distant taxa is consistent with a broad evolutionary distribution of this channel family among bilaterian animals.

In contrast to the diversification observed for TRPA channels, TRPN was recovered as a single phylogenetic group in the taxa examined here, with no evidence of major lineage-specific expansions. Detailed information regarding sequence accession numbers and phylogeny-based annotations is provided in **Supplementary Table 1** and **Supplementary Figure 6**.

#### 3.1.3 TRPV phylogeny

**Figure 4:**
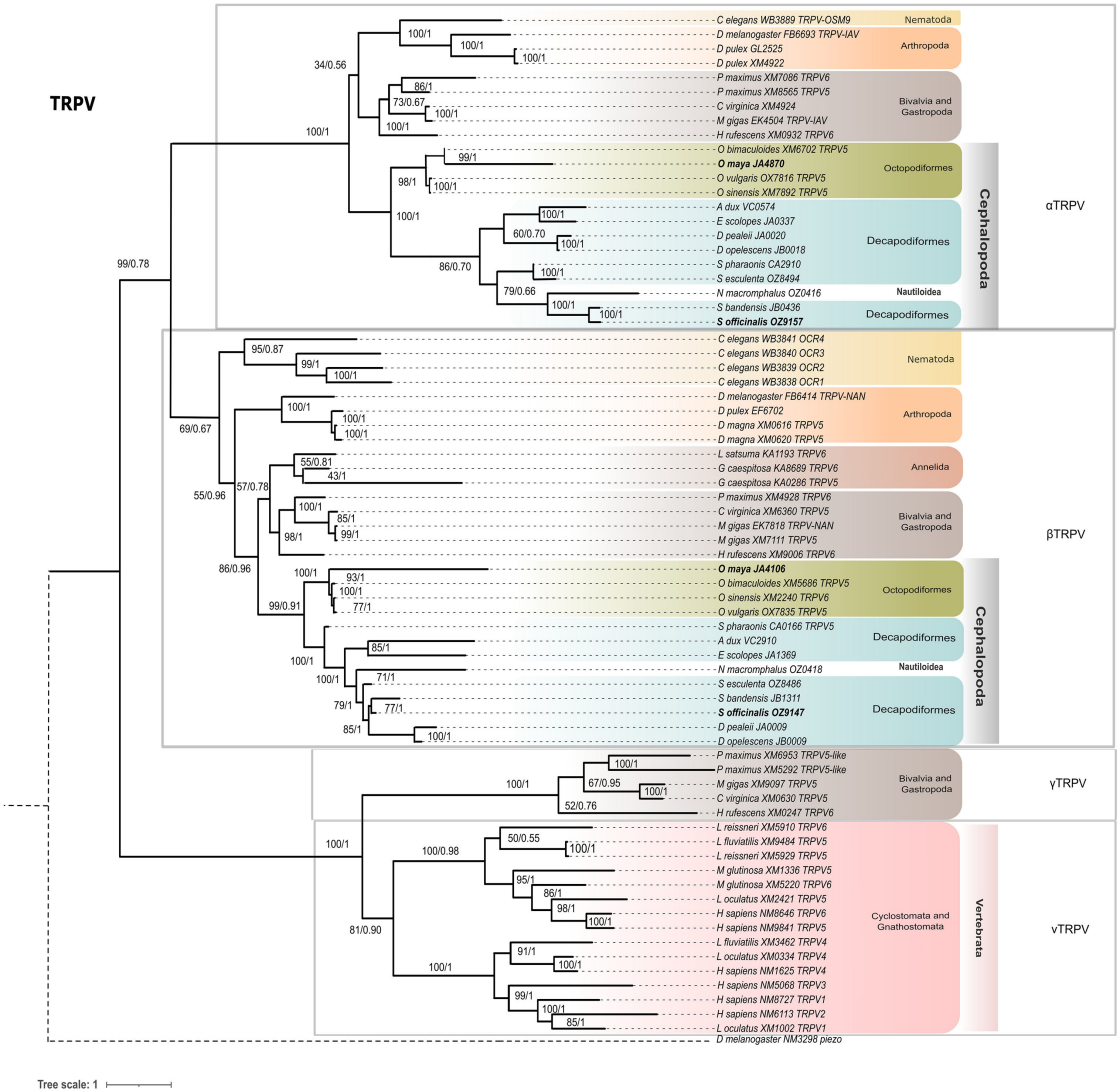
Detailed phylogeny of the TRPV channel family. The subtree recovered four principal TRPV groups, designated αTRPV, βTRPV, γTRPV, and νTRPV. *Sepia officinalis* and *Octopus maya* sequences are highlighted in bold. Support values are shown at the nodes.

**Figure 4** highlights the phylogeny relationships within the TRPV (Vanilloid) channel family based on 71 amino acid sequences from representative vertebrate and non-vertebrate taxa, including a broad sampling of cephalopods. The analyses reveals four principal TRPV clades that were consistently inferred by both RAxML and MrBayes. Three of these clades were composed predominantly of non-vertebrate sequences and are here designated αTRPV, βTRPV, and γTRPV, whereas the fourth clade comprised vertebrate TRPV sequences, including TRPV1–TRPV6 homologues from gnathostomes (*Homo sapiens* and *Lepisosteus oculatus*) together with related TRPV homologues from cyclostomes (*Lampetra fluviatilis*, *Lethenteron reissneri*, and *Myxine glutinosa*). Support values for the nodes defining these major clades ranged from moderate to high (αTRPV: BS = 73, PP = 0.77; βTRPV: BS = 100, PP = 1.00; γTRPV: BS = 100, PP = 1.00; vertebrate TRPV: BS = 81, PP = 0.90) (**Figure 4**).

The αTRPV clade encompasses sequences from a broad range of metazoan taxa. Within cephalopods, αTRPV sequences were identified from representatives of Decapodiformes (e.g., *Sepia officinalis*, *S. bandensis, Doryteuthis pealeii*), Octopodiformes (e.g., *Octopus maya*, *O. bimaculoides*), as well as Nautiloidea (*Nautilus macromphalus*). Outside cephalopods, this clade includes homologues from bivalves (e.g., *Magallana gigas*, *Crassostrea virginica*), gastropods (e.g., *Haliotis rufescens*), annelids (e.g., *Galeolaria caespitosa*), arthropods (e.g., *Drosophila melanogaster*, *Daphnia pulex*), and the nematode *Caenorhabditis elegans*. The presence of the well-characterised arthropod TRPV-IAV channel (*D. melanogaster*) and the TRPV-OSM-9 channel (*C. elegans*) within αTRPV clade is consistent with a broad distribution of this group among protostome taxa. This αTRPV subtype corresponds to the TRPVA clade in the nomenclature by Morini et al. (2022).

The βTRPV clade was identified as the sister group to αTRPV and contains sequences representatives from arthropods, molluscs, nematodes, and cephalopods. Within cephalopods, βTRPV sequences were identified in Decapodiformes, Octopodiformes and Nautiloidea. The occurrence of βTRPV homologues in representatives of these three cephalopod lineages indicates a broad distribution of this group among the cephalopod taxa examined here. In addition to cephalopods, this clade includes homologues from bivalves (e.g., *Magallana gigas, Pecten maximus*) and gastropods (e.g., *Haliotis rufescens*). Notably, several well-characterised protostomian TRPV channels, including TRPV-NAN from the fruit fly *D. melanogaster* and TRPV-OCR-1 to OCR-4 from the nematode *C. elegans*, were also recovered within this clade. This βTRPV subtype corresponds to the TRPVB clade in the nomenclature by Morini et al. (2022).

The γTRPV clade forms a distinct group composed exclusively of molluscan sequences. Members of this clade include homologues from bivalves (e.g., *Crassostrea virginic*, *Pecten maximus*) and the gastropod *(Haliotis rufescens)*. Notably, no cephalopod sequences were recovered within γTRPV in the present analysis.

The vertebrate TRPV clade comprises homologues from cyclostomes (*L. fluviatilis*, *L. reissneri*, and *M. glutinosa*) and gnathostomes (*Homo sapiens* and *Lepisosteus oculatus*). This clade includes representatives corresponding to TRPV1–TRPV6 and is clearly separated from the αTRPV, βTRPV, and γTRPV groups recovered in non-vertebrate taxa.

Several non-vertebrate sequences originally annotated as TRPV5/5-like or TRPV6/6-like consistently groups within the αTRPV, βTRPV, or γTRPV clades, rather than with vertebrate TRPV5 or TRPV6 homologues. Based on the phylogenetic relationships inferred from both RAxML and MrBayes analyses, we propose the use of the designations αTRPV, βTRPV, and γTRPV to distinguish the three principal non-vertebrate TRPV clades identified in the present dataset.

Detailed information on the sequences of TRPV channels and our proposed phylogenetically-based annotations is presented in **Supplementary Table 1** and **Supplementary Figure 7**.

*TRPC phylogeny*.

**Figure 5:**
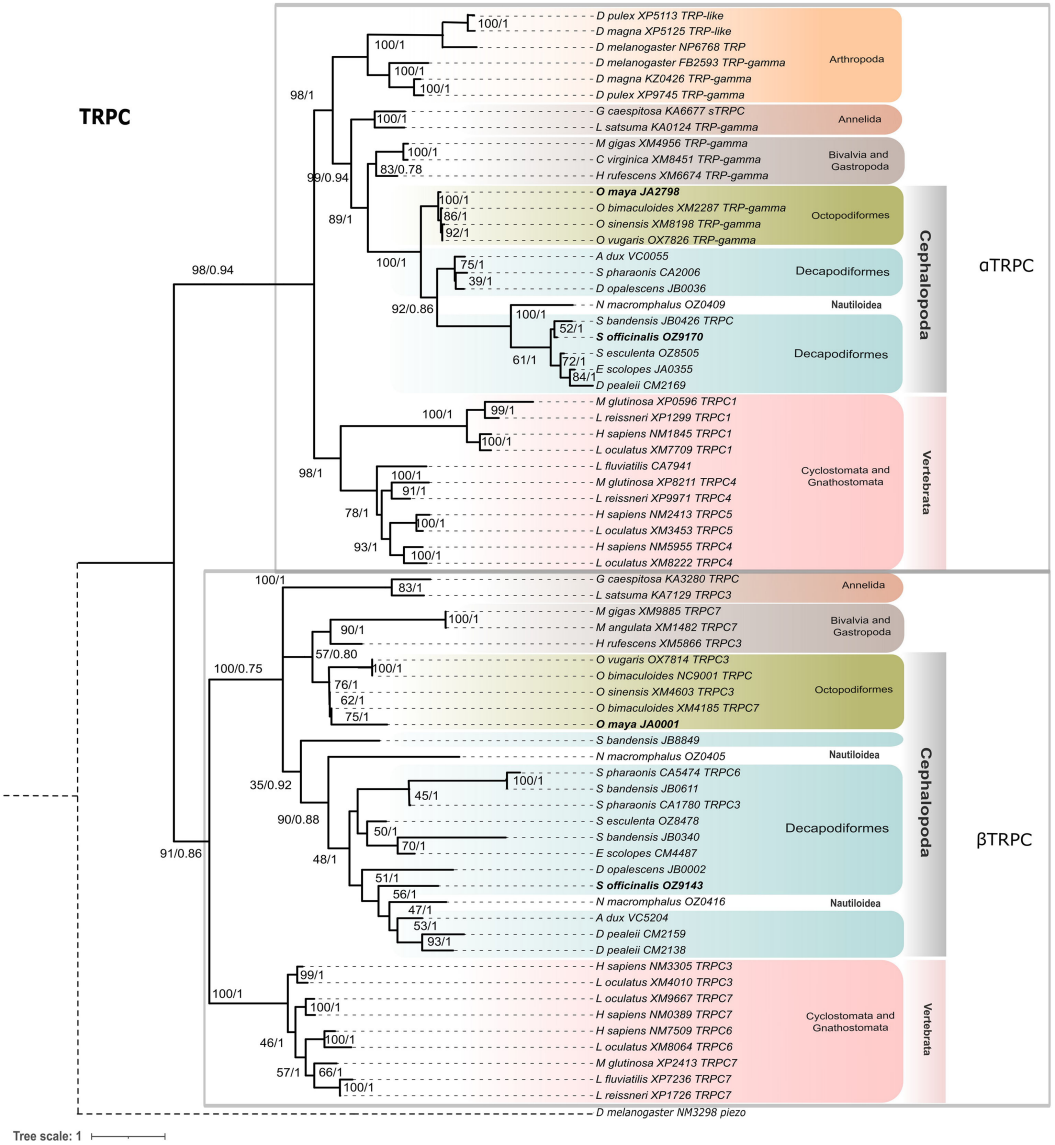
Detailed phylogeny of the TRPC channel family. The subtree recovered two principal TRPC groups, designated αTRPC and βTRPC. *Sepia officinalis* and *Octopus maya* sequences are highlighted in bold. Support values are shown at the nodes.

**Figure 5** highlights the phylogenetic relationships among 68 TRPC (Canonical) channels sequences from representative vertebrate and invertebrate taxa, including a broad sampling of cephalopods. The analyses recovered two principal TRPC clades, designated αTRPC and βTRPC. The phylogeny recovered two major TRPC clades, designated αTRPC and βTRPC, which were consistently identified by both RAxML and MrBayes analyses. The basal nodes defining these clades were supported by both phylogenetic approaches (αTRPC: BS = 98, PP = 0.94; βTRPC: BS = 91, PP = 0.86).

The αTRPC clade encompasses sequences from a broad range of metazoan taxa, including arthropods, annelids, molluscs, cephalopods, and vertebrates. Within cephalopods, αTRPC sequences were recovered from representatives of Octopodiformes (e.g., *Octopus maya, O. bimaculoides, O. vulgaris*), Decapodiformes (e.g., *Sepia officinalis, S. bandensis, Doryteuthis pealeii*) and Nautiloidea (*Nautilus macromphalus*). Outside cephalopods, this clade includes homologues from bivalves (e.g., *Magallana gigas, Crassostrea virginica*), the gastropod (e.g., *Haliotis rufescens*), annelids (*Galeolaria caespitosa* and *Lamellibrachia satsuma*), arthropods (*Drosophila melanogaster*, *Daphnia magna*, and *D. pulex*), and vertebrates represented by cyclostomes (*Myxine glutinosa* and *Lethenteron reissneri*) and gnathostomes (*Homo sapiens* and *Lepisosteus oculatus*).

Several non-vertebrate sequences previously annotated as TRP-gamma, including those from *Octopus bimaculoides*, *Magallana gigas*, and *Drosophila melanogaster*, consistently recovered within the αTRPC clade. In addition, numerous unannotated sequences, particularly from Decapodiformes (e.g., *Sepia officinalis*) and Octopodiformes (e.g., *Octopus maya*), were also placed within the αTRPC clade, supporting their putative orthology with the previously annotated TRP-gamma. This clade also includes well-characterised vertebrate TRPC1, TRPC4 and TRPC5 channels sequences.

The βTRPC clade likewise exhibits broad taxonomic representation and includes sequences from annelids, molluscs, cephalopods, and vertebrates. Within cephalopods, βTRPC homologues were recovered from Octopodiformes, Decapodiformes, and Nautiloidea. The recovery of βTRPC sequences from these three cephalopod groups is consistent with the occurrence of this lineage across the cephalopod taxa sampled in the present study. Outside cephalopods, βTRPC includes homologues from bivalves (*Magallana gigas* and *M. angulata*), the gastropod *Haliotis rufescens*, annelids (*Galeolaria caespitosa* and *Lamellibrachia satsuma*), and vertebrates represented by both cyclostomes and gnathostomes.

Several non-vertebrate sequences originally annotated as TRPC3, TRPC6 or TRPC7, including those from *Sepia pharaonis*, *Octopus bimaculoides*, and *Magallana gigas*, were placed within the βTRPC clade. In addition, numerous previously unannotated sequences, particularly from Decapodiformes (e.g., *Sepia officinalis*) and Octopodiformes (e.g., *Octopus maya*), were also encompassed within the βTRPC clade, suggesting that they may represent additional members of the βTRPC subtype. This clade further encompasses well-characterised vertebrate TRPC3, TRPC6, and TRPC7 homologues.

As observed for the αTRPC clade, the recovery of βTRPC homologues from both protostome and deuterostome taxa is consistent with a broad phylogenetic distribution of this subtype. The previous annotations of several non-vertebrate sequences as TRPC3, TRPC6, or TRPC7 Proteins is consistent with their placement within the βTRPC clade together with vertebrate TRPC3, TRPC6, and TRPC7 homologues. Nevertheless, the phylogenetic relationships among these sequences do not allow an unambiguous assignment of non-vertebrate homologues to specific vertebrate TRPC subtypes. For this reason, we consider the designation of these sequences as βTRPC homologues to be the most conservative interpretation of the present dataset.

Within vertebrates, TRPC diversity is distributed across both αTRPC and βTRPC. Vertebrate TRPC1, TRPC4, and TRPC5 homologues were recovered within αTRPC, whereas TRPC3, TRPC6, and TRPC7 homologues were recovered within βTRPC. A similar pattern was observed in cephalopods, where representatives of both αTRPC and βTRPC were identified in Octopodiformes, Decapodiformes, and Nautiloidea. Furthermore, several cephalopod species were found to possess multiple sequences, particularly within βTRPC clade, including *Sepia bandensis*, *S. pharaonis*, *Doryteuthis pealeii*, and *Octopus bimaculoides*. The presence of multiple βTRPC sequences in these species may be consistent with lineage-specific duplication events, although additional analyses would be required to confirm their evolutionary origin.

The recovery of previously annotated TRP-gamma sequences together with vertebrate TRPC1/4/5 homologues in αTRPC, and of previously annotated TRPC3/6/7-like sequences together with vertebrate TRPC3/6/7 homologues in βTRPC, supports the recognition of two principal TRPC groups across the taxa examined here. Based on the phylogenetic relationships inferred from both RAxML and MrBayes analyses, we propose the use of the designations αTRPC and βTRPC to distinguish these two major TRPC clades.

Detailed information on the sequences of TRPC channels and our proposed phylogenetically-based annotations is presented in **Supplementary Table 1** and **Supplementary Figure 8**.

#### 3.1.4 TRPML phylogeny

**Figure 6:**
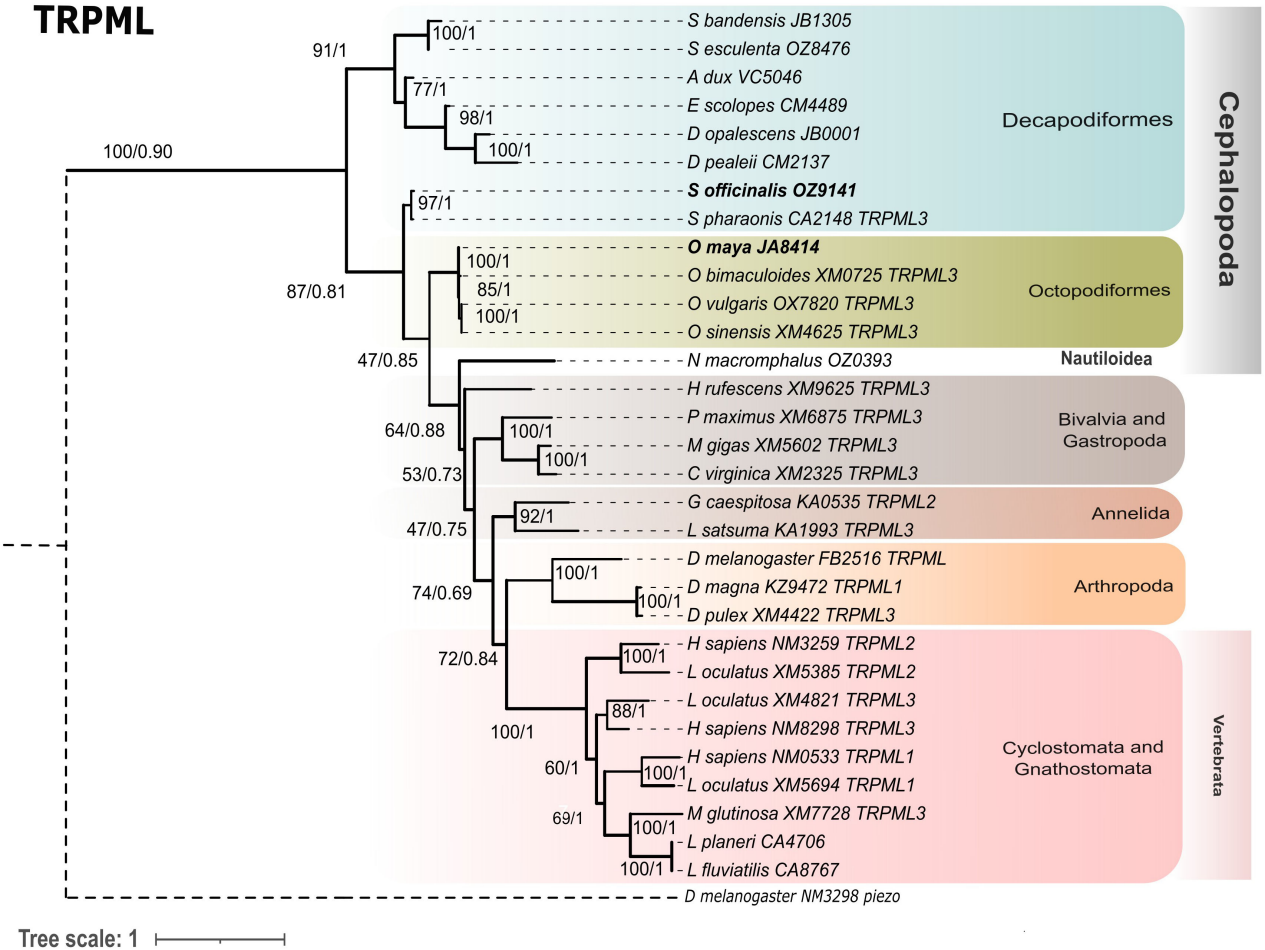
Detailed phylogeny of the TRPML channel family. The subtree identified a single principal TRPML clade. *Sepia officinalis* and *Octopus maya* sequences are highlighted in bold. Support values are shown at the nodes.

The phylogenetic tree in **Figure 6** highlights the phylogenetic relationships within the TRPML (Mucolipin) channel family based on 31 amino acid sequences from representative vertebrate and non-vertebrate taxa, including a broad sampling of cephalopods. In contrast to the TRPA, TRPV, and TRPC families, the analyses recovered a single principal TRPML clade that was consistently inferred by both RAxML and MrBayes (BS = 100, PP = 0.90) (**Figure 6**).

The TRPML clade encompasses sequences from a broad range of metazoan taxa, including cephalopods, other molluscs, annelids, arthropods, and vertebrates. Within cephalopods, TRPML sequences were recovered from representatives of Decapodiformes (e.g., *Sepia officinalis*, *Doryteuthis pealeii*, *Architeuthis dux*), Octopodiformes (e.g., *Octopus maya*, *O. vulgaris*), and Nautiloidea (*Nautilus macromphalus*). Outside cephalopods, the TRPML clade includes homologues from bivalves (e.g., *Magallana gigas, Crassostrea virginica, and Pecten maximus*), the gastropod *Haliotis rufescens*, annelids (*Galeolaria caespitosa* and *Lamellibrachia satsuma*), arthropods (*Drosophila melanogaster*, *Daphnia magna*, and *D. pulex*), and vertebrates represented by cyclostomes (*Lampetra planeri*, *L. fluviatilis*, and *Myxine glutinosa*) and gnathostomes (*Homo sapiens* and *Lepisosteus oculatus*).

Several non-vertebrate sequences originally annotated as TRPML3 or MCOLN3, were recovered within this clade together with numerous previously unannotated cephalopod sequences. Likewise, vertebrate TRPML1, TRPML2, and TRPML3 homologues were also recovered within the same principal group. The recovery of TRPML homologues in representatives of Octopodiformes, Decapodiformes, and Nautiloidea indicates that this channel family is broadly distributed among the cephalopod taxa examined here.

The inclusion of the *Drosophila melanogaster* TRPML homologue within this clade further illustrates the wide taxonomic representation of TRPML sequences recovered in the present analysis.

Based on the phylogenetic relationships inferred from both RAxML and MrBayes analyses, we retained the designation TRPML for all sequences included in this clade. Detailed information on the sequences of TRPML channels and our proposed phylogenetically-based annotations is presented in **Supplementary Table 1** and **Supplementary Figure 9**.

#### 3.1.5 TRPP phylogeny

**Figure 7:**
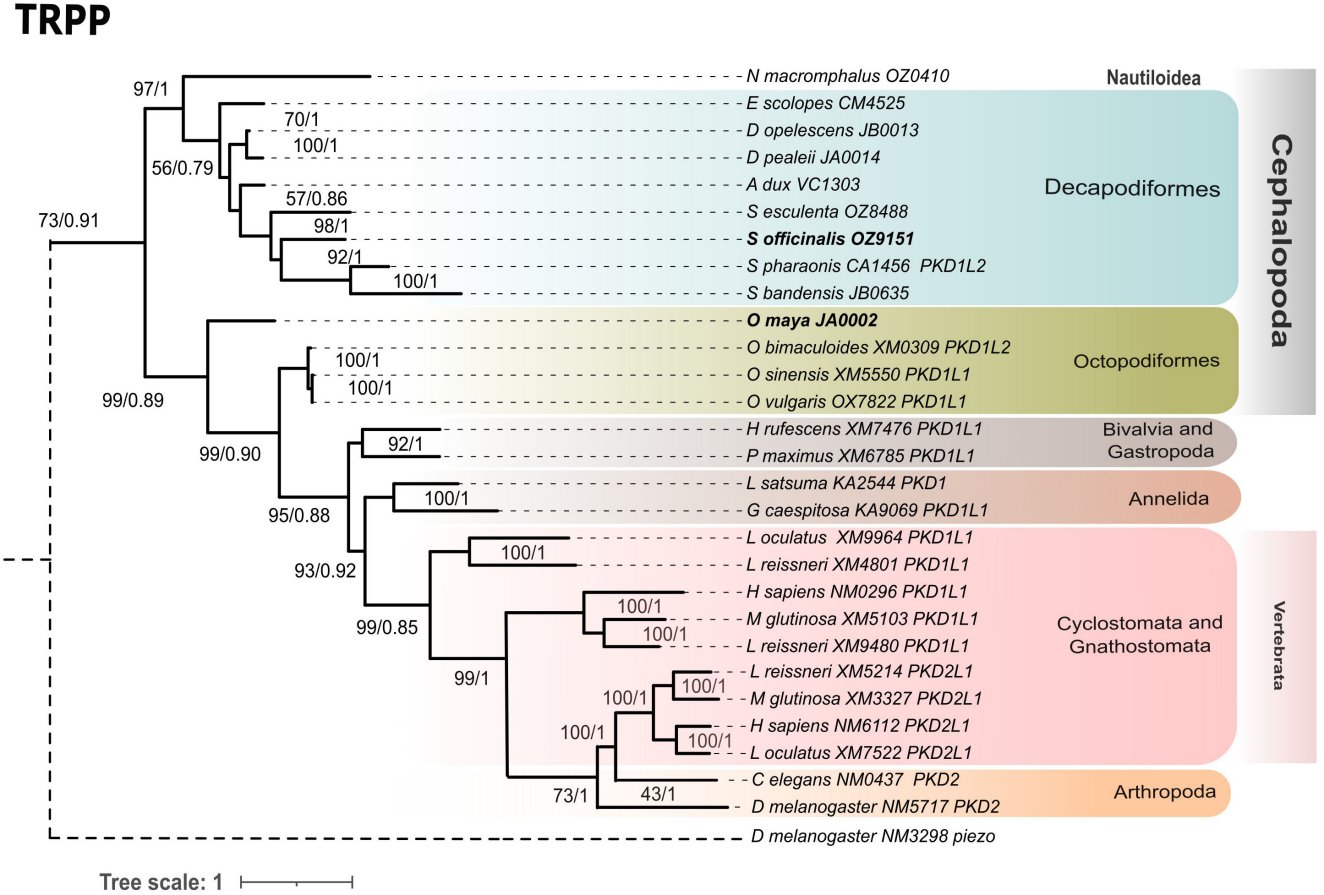
Detailed phylogeny of the TRPP channel family. The subtree revealed a single principal TRPP clade. *Sepia officinalis* and *Octopus maya* sequences are highlighted in bold. Support values are shown at the nodes.

**Figure 7** highlights the phylogenetic relationships within the TRPP (Polycystin; PKD) channel family based on 28 amino acid sequences from representative vertebrate and non-vertebrate taxa, including a broad sampling of cephalopods. The analyses recovered a principal TRPP clade that was consistently inferred by both RAxML and MrBayes (BS = 73, PP = 0.91) (**Figure 7**).

Within vertebrates, TRPP sequences were resolved into two distinct subgroups corresponding to PKD1/PKD1L1/2 (*alias* TRPP1) and PKD2/PKD2L1 (*alias* TRPP2) homologues. These groups included representatives of gnathostomes (*Homo sapiens* and *Lepisosteus oculatus*) together with cyclostomes (*Myxine glutinosa* and *Lethenteron reissneri*). In addition, the PKD2 homologues of *Drosophila melanogaster* and *Caenorhabditis elegans* were recovered in association with the vertebrate PKD2/PKD2L1 group.

The non-vertebrate TRPP clade encompasses sequences from cephalopods, other molluscs, and annelids. Within cephalopods, TRPP sequences were recovered from representatives of Decapodiformes (e.g., *Sepia officinalis*, *Euprymna scolopes*), Octopodiformes (e.g., *Octopus vulgaris*, *O. maya*), and Nautiloidea (*Nautilus macromphalus*). The recovery of TRPP homologues in representatives of these three cephalopod lineages indicates a broad distribution of this channel family among the cephalopod taxa examined here.

Outside cephalopods, this clade includes homologues from bivalves (*Pecten maximus*), the gastropod *Haliotis rufescens*, and annelids (*Galeolaria caespitosa* and *Lamellibrachia satsuma*). Several non-vertebrate sequences previously annotated as PKD1L1 or PKD1L2 were recovered within this clade together with numerous previously unannotated cephalopod sequences. In contrast to vertebrates, where PKD1/PKD1L1 and PKD2/PKD2L1 formed distinct subgroups, the non-vertebrate sequences included in the present analysis were not resolved into comparable subdivisions.

The phylogenetic placement of the molluscan and cephalopod sequences as a sister lineage to the vertebrate PKD1/PKD1L1 and PKD2/PKD2L1 clades does not, on its own, provide sufficient support for assigning these sequences to either vertebrate subgroup. Accordingly, we retained the designation TRPP for the non-vertebrate sequences recovered in the present study.Detailed information on the sequences of TRPP channels and our proposed phylogenetically-based annotations is presented in **Supplementary Table 1** and **Supplementary Figure 9**.

#### 3.1.6 TRPM phylogeny

**Figure 8:**
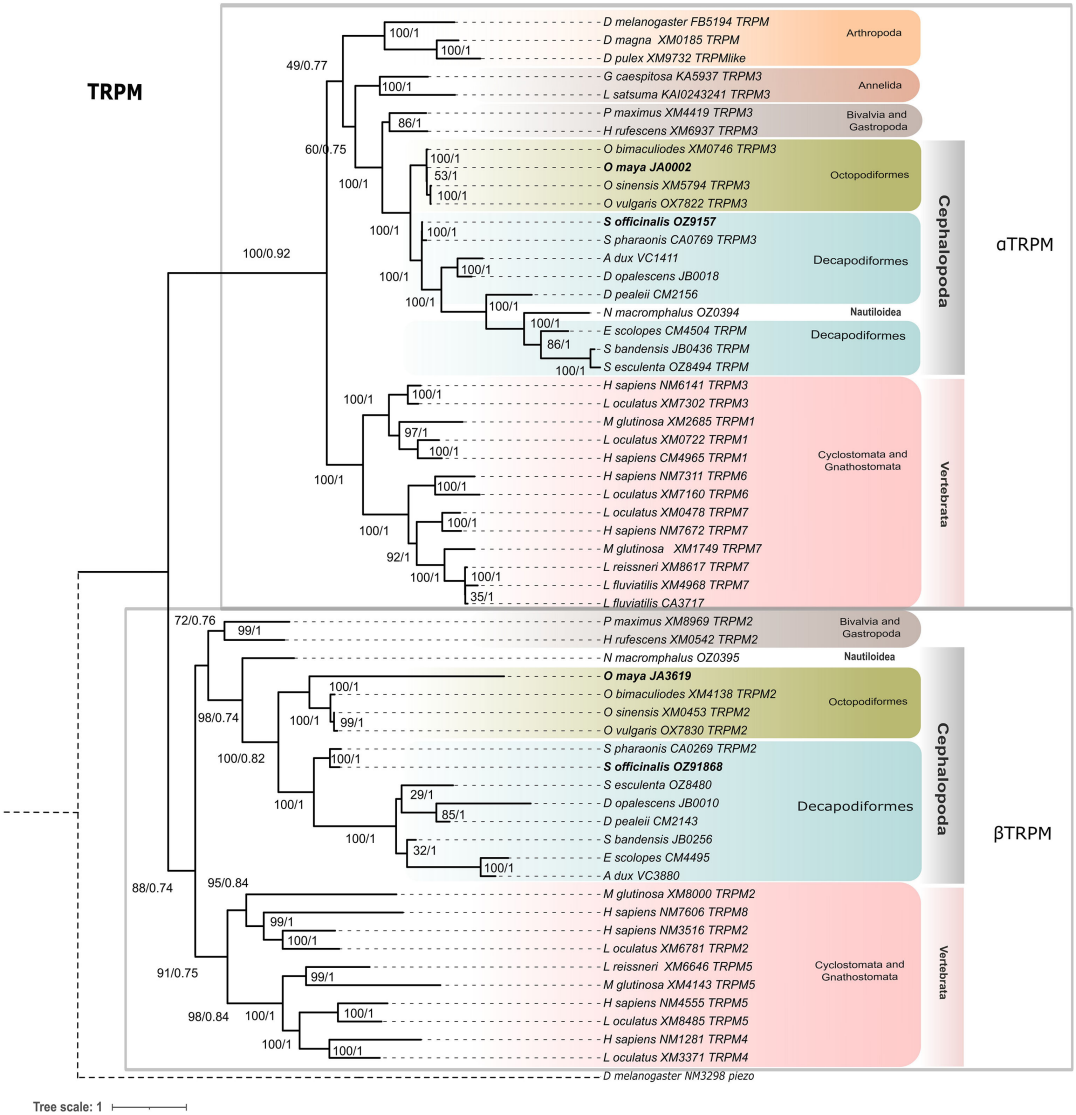
Detailed phylogeny of the TRPM channel family. The subtree resolved two principal TRPM groups, designated αTRPM and βTRPM. *Sepia officinalis* and *Octopus maya* sequences are highlighted in bold. Support values are shown at the nodes.

**Figure 8** highlights the phylogenetic relationships among 58 TRPM (Melastatin) channel sequences based on amino acid sequences from representative vertebrate and non-vertebrate taxa, including a broad sampling of cephalopods (**Supplementary Table 1**). The analyses identified two principal TRPM clades, designated αTRPM and βTRPM, which were consistently recovered by both RAxML and MrBayes. The nodes defining these clades were associated with moderate to high bootstrap values and posterior probabilities (αTRPM: BS = 100, PP = 0.92; βTRPM: BS = 88, PP = 0.74) (**Figure 8**).

The αTRPM clade encompasses sequences from a broad range of metazoan taxa, including arthropods, annelids, molluscs, cephalopods, and vertebrates. Within cephalopods, αTRPM sequences were recovered from representatives of Decapodiformes (e.g., *Sepia officinalis*, *Doryteuthis pealeii*, *Architeuthis dux*), Octopodiformes (e.g., *Octopus maya*, *O. vulgaris*), and Nautiloidea (*Nautilus macromphalus*). Outside cephalopods, this clade includes homologues from bivalves (*Pecten maximus*), the gastropod *Haliotis rufescens*, annelids (*Galeolaria caespitosa* and *Lamellibrachia satsuma*), arthropods (*Drosophila melanogaster*, *Daphnia magna*, and *D. pulex*). Several non-vertebrate sequences originally annotated as TRPM3, including those from *Sepia pharaonis*, *Octopus bimaculoides*, and *Pecten maximus*, consistently placed within the αTRPM clade. In addition, numerous previously unannotated sequences, particularly from Decapodiformes (e.g., *Sepia officinalis*), Octopodiformes (e.g., *Octopus maya*), and Nautiloidea (*N. macromphalus*) likewise within the αTRPC clade. Among non-vertebrates, the *Drosophila melanogaster* TRPM sequence also encompassed within this clade. The αTRPM clade also contains vertebrate TRPM1, TRPM3, TRPM6, and TRPM7 homologues.

The βTRPM clade likewise exhibits broad taxonomic representation and includes sequences from molluscs, cephalopods, and vertebrates. Within cephalopods, βTRPM homologues were recovered from Octopodiformes, Decapodiformes, and Nautiloidea, including *Nautilus macromphalus.* The occurrence of βTRPM sequences in representatives of these three cephalopod lineages indicates a broad distribution of this group among the cephalopod taxa examined here. Outside cephalopods, this clade includes homologues from bivalves (*Pecten maximus*), the gastropod *Haliotis rufescens*, and vertebrates represented by cyclostomes and gnathostomes. Several non-vertebrate sequences previously annotated as TRPM2 were recovered within βTRPM together with numerous previously unannotated cephalopod sequences. This clade also contains vertebrate TRPM2, TRPM4, TRPM5, and TRPM8 homologues.

Within vertebrates, TRPM diversity is distributed across both αTRPM and βTRPM. Vertebrate TRPM1, TRPM3, TRPM6, and TRPM7 homologues were recovered within αTRPM, whereas vertebrate TRPM2, TRPM4, TRPM5, and TRPM8 homologues were recovered within βTRPM. A similar pattern was observed in cephalopods, where representatives of both αTRPM and βTRPM were identified in Octopodiformes, Decapodiformes, and Nautiloidea. The recovery of αTRPM and βTRPM sequences in representatives of all major cephalopod lineages included in this study indicates that both groups are broadly distributed among extant cephalopods.

Based on the phylogenetic relationships inferred from both RAxML and MrBayes analyses, we propose the use of the designations αTRPM and βTRPM to distinguish the two principal TRPM clades identified in the present dataset. Detailed information regarding sequence accession numbers and phylogeny-based annotations is provided in **Supplementary Table 1**.

Detailed information on the sequences of TRPM channels and our proposed phylogenetically-based annotations is presented in **Supplementary Table 1** and **Supplementary Figure 10**.

### 3.2 TRP channel repertoire in cephalopods

The phylogenetic relationships inferred in the present analyses provided a framework for assigning candidate sequences to the major TRP (Transient Receptor Potential) channel families. As summarised in **Table 1**, we analysed the diversity of 117 previously unannotated and 41 previously annotated TRP channel sequences across 13 cephalopod species. Notably, each species possesses representatives of all seven major TRP families (TRPA, TRPN, TRPV, TRPC, TRPM, TRPML and TRPP), along with their respective subtypes (α/β/γ), revealed in the present study, indicating that the core TRP channel repertoire is conserved across cephalopod lineages.

**Table 1:** Candidates transient receptor potential (TRP) channels in cephalopods.

Species Name			TRPA		TRPC		TRPM		TRPV		TRPML	TRPP	TRPN		
		αTRP A	βTRP A	γTRP A	αTRP C	βTRP C	αTRPM	βTRP M	αTRP V	βTRP V	TRPML	TRPP	TRPN	Total	
***Sepia officinalis***			1	1	1	1	1	1	1	1	1	1	1	11	
*Acanthosepion pharaonis (Sepia* *pharaonis)*			[1]	1	1	[2]	[1]	[1]	[1]	[1]	[1]	[1]	1	12	
*Acanthosepion esculentum (Sepia* *esculenta)*			1	1	1	1	1	1	1	1	1	1	1	11	
*Ascarosepion bandense (Sepia* *bandensis)*			1	1	1	3	1	1	1	1	1	1	1	13	
***Octopus maya***			1	1	1	1	1	1	1	1	1	1	1	11	
*Octopus vulgaris*		[1]	[1]	1	[1]	[1]	[1]	[1]	[1]	[1]	[1]	[1]	1	12	
*Octopus sinensis*		[1]	[1]	1	[1]	[1]	[1]	[1]	[1]	[1]	[1]	[1]	1	12	
*Octopus bimaculoides*		[2]	[1]	1	[1]	1[1]	[1]	[1]	[1]	[1]	[1]	[1]	1	14	
*Doryteuthis pealeii*				2	1	2	1	1	1	1	1	1	1	13	
*Doryteuthis opalescens*			1	4	1	1	1	1	1	1	1	1	1	14	
*Architeuthis dux*			1	1	1	1	1	1	1	1	1	1	1	11	
*Euprymna scolopes*			1	1	1	1	1	1	1	1	1	1	1	11	
*Nautilus macromphalus*			1	1	1	2	1	1	1	1	1	1	1	12	
**Total**														**157**	

Numbers in brackets [] indicate previously annotated sequences retrieved from public databases for each corresponding species. See **Supplementary Table 1** for further details.

The number of TRP channel sequences exhibits interspecific variation, ranging from 11 to 14 per species. In several species, including *Doryteuthis pealeii*, *D. opalescens*, *Octopus bimaculoides*, *Sepia esculenta and Nautilus macromphalus*, paralogs were identified within specific TRP families and subtypes (α and γTRPA and βTRPC), potentially reflecting lineage- or species-specific gene duplication events. Detailed information regarding TRP sequences, including proposed nomenclature, protein/gene accession numbers, chromosomal locations, and sequence characteristics, is provided in **Supplementary Table 1**.

### 3.3 Effects of temperature on embryonic development

In *Sepia officinalis*, embryos exposed to 22 °C reached stage 30 after approximately 20 days of incubation, whereas embryos maintained at 16 °C required approximately 44 days to reach the same developmental stage. These observations indicate a substantially shorter developmental period at 22 °C, with embryos reaching hatching nearly twice as fast as those maintained at 16 °C following exposure from stages 23–24. No obvious morphological abnormalities were observed in embryos from either temperature treatment.

Similarly, in *Octopus maya*, embryos incubated at 30 °C reached stage 19 after approximately 30 days, whereas embryos maintained at 24 °C required approximately 45 days to reach the same developmental stage. These observations indicate a shorter developmental period under elevated temperature conditions, consistent with the pattern observed in *S. officinalis*. No externally detectable abnormalities were observed in embryos maintained at 24 °C. Although developmental abnormalities have previously been reported in *O. maya* embryos exposed to 30 °C, including mantle, arm, and ocular defects (Caamal-Monsreal et al., 2016; Galindo-Torres et al., 2024), only embryos without externally detectable abnormalities were selected for subsequent gene expression analyses.

### 3.4 Expression analysis of candidate TRP channels in whole embryos of *S. officinalis* and *O. maya* under optimal and stress conditions

**Figure 9:**
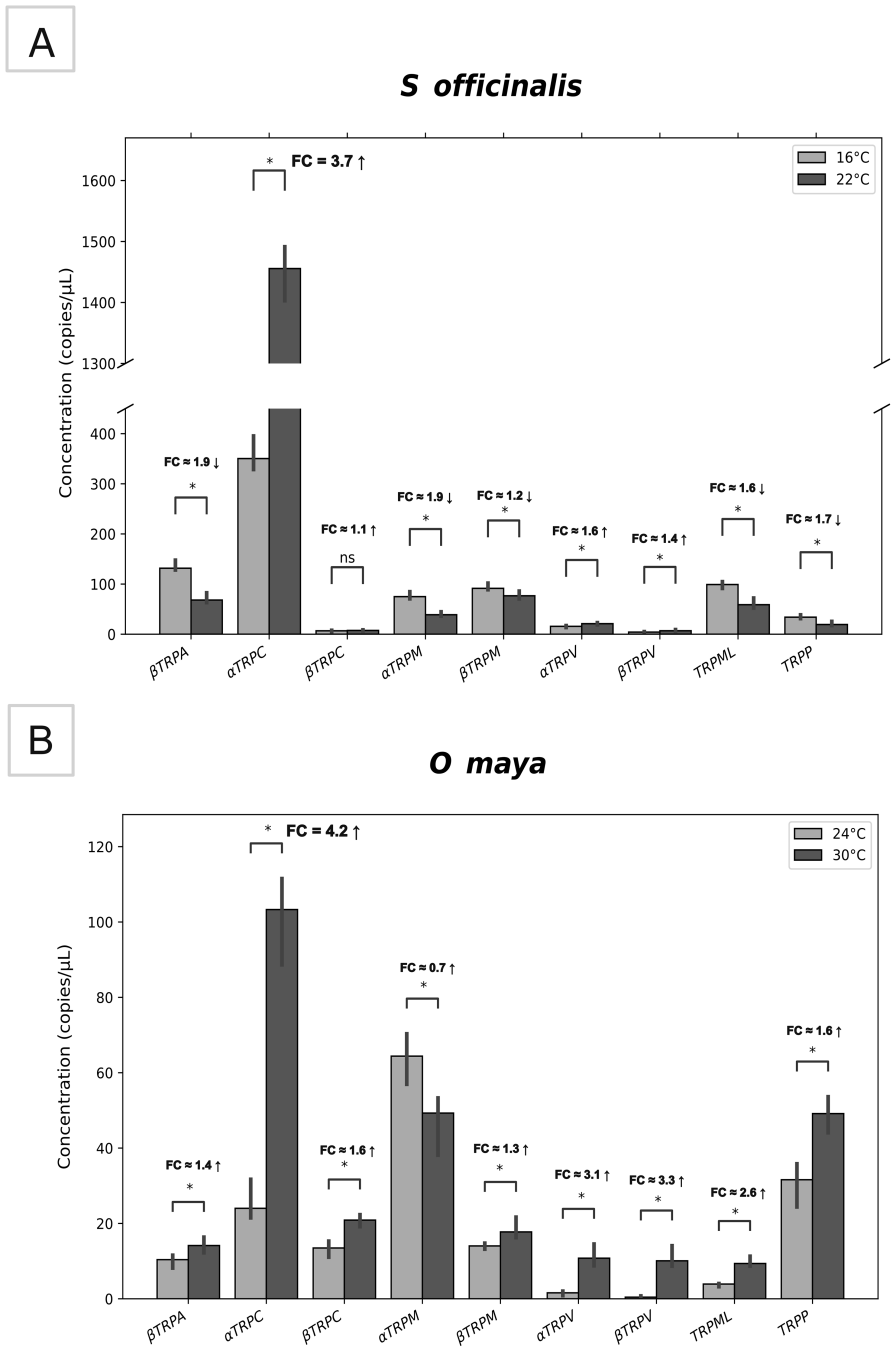
Gene expression levels (copies/μL) of the TRP sequences (βTRPA, αTRPC, βTRPC, αTRPM, βTRPMB, αTRPV, βTRPV, TRPML, and TRPP) in whole cephalopod embryos. **(A)** *S. officinalis* embryos (stage 30, before hatching) after 20 days of chronic exposure to both optimal (16 °C) and heat stress (22 °C) temperatures. **(B)** *O. maya* embryos (stage 30, before hatching) after 30 days of chronic exposure to both optimal (24 °C) and heat stress (30 °C) temperatures. Bars represent mean ± SEM (n = 5 embryos per temperature). For each gene, temperatures were compared using a two-sided permutation test, followed by Benjamini–Hochberg FDR correction across genes. Statistical significance was set at q < 0.05. FC denotes fold change (heat stress/optimal) computed from means on the original scale. * indicates statistically significant differences (q < 0.05).

To explore the thermal responsiveness of TRP channel expression, we analysed their transcript levels in whole embryos of two ecologically and economically relevant cephalopod species, a decapodiforme *Sepia officinalis*, and an octopodiforme *Octopus maya*. We developed digital PCR assays for nine TRP channels in each species (**Supplementary Table 3**). Our findings revealed a combination of conserved and species-specific expression patterns across the TRP channels analysed (**Figure 9**).

Among the transcripts examined, αTRPC displayed the strongest and most consistent upregulation under thermal stress conditions in both species, with significant increases observed in *S. officinalis* (3.7-fold) and *O. maya* (4.2-fold). Under thermal stress conditions, αTRPC also reached comparatively higher expression level relative to the other TRP transcripts analysed in both species, although absolute expression levels were markedly higher in *S. officinalis* that in *O. maya*. In addition, αTRPV expression was significantly upregulated under thermal stress in both species (~1.6-fold in *S. officinalis*; ~3.1-fold in *O. maya*), although its expression levels and magnitude of induction remained lower than those observed for αTRPC (**Figure 9**).

In contrast, species-specific responses were observed in the regulation of other TRP channels. Opposing regulatory responses to heat stress were detected for TRPML and TRPP: both of which were significantly downregulated in *S. officinalis*, whereas they were upregulated in *O. maya*. Similarly, βTRPA and αTRPM were markedly downregulated in *S. officinalis* under thermal stress, while their expression remained unchanged in *O. maya.* Conversely, in *O. maya*, the expression of βTRPC and βTRPV was significantly upregulated in response to elevated temperature, whereas no changes were detected for these genes in *S. officinalis*. Lastly, βTRPM expression did not significantly differ between treatments in either species (**Figure 9**).

Taken together, these finding suggest that αTRPC may represent a promising candidate temperature-responsive marker in cephalopods, given its relatively high expression levels and significant upregulation under elevated temperatures in both *S. officinalis* and *O. maya*.

### 3.5 Expression analysis of TRP channels in different tissues of *S. officinalis* and *O. maya* under optimal and heat stress conditions

**Figure 10:**
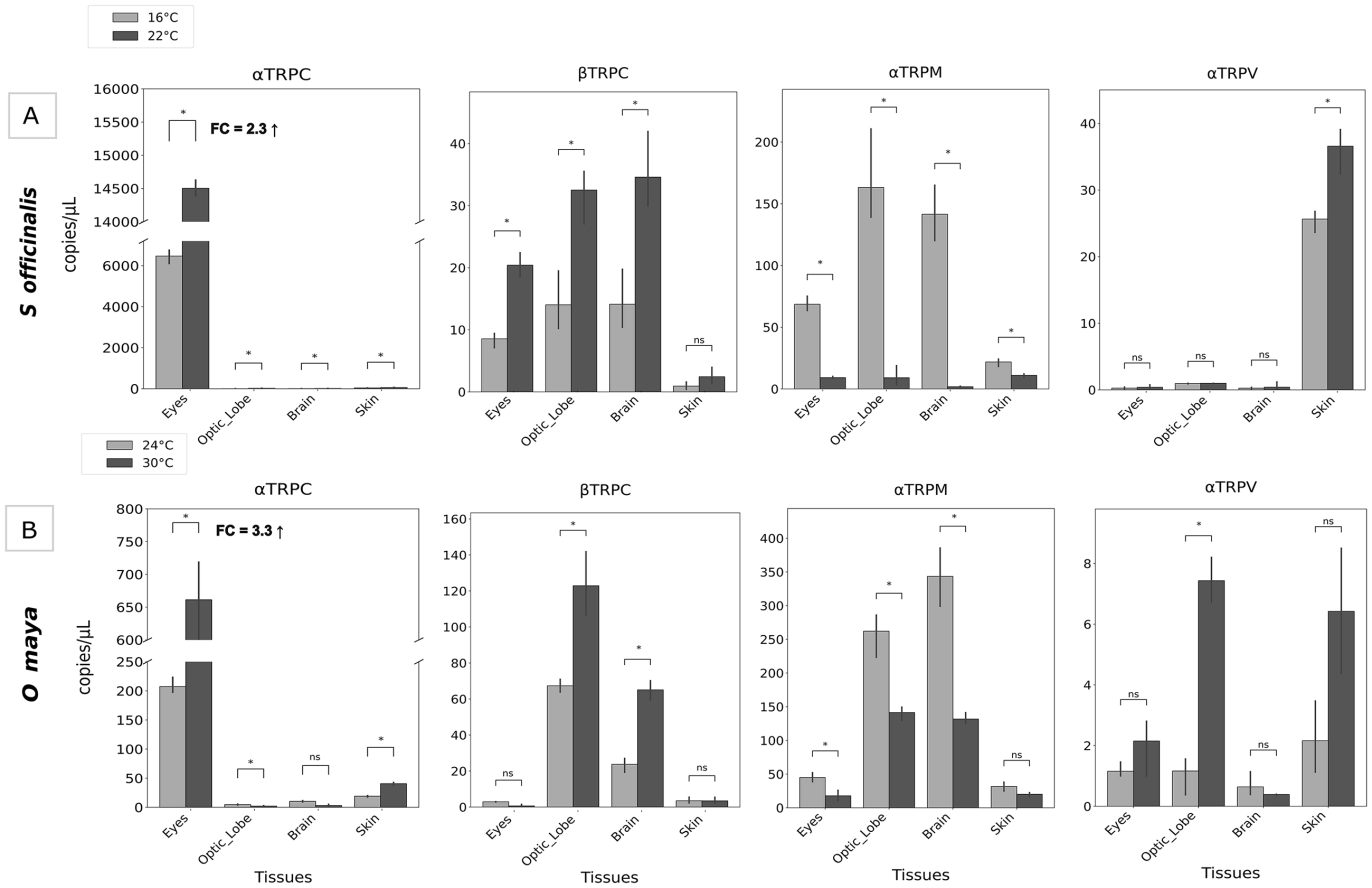
Expression profile of selected TRP channels (copies/μL) across four distinct tissues (eyes, optic lobes, brain and skin) in *S. officinalis* and *O. maya* embryos exposed to heat temperature stress. **(A)** Gene expression profile in *S. officinalis* after 20 days of chronic exposure to optimal (16 °C) and heat stress (22 °C) temperatures. **(B)** Gene expression profile in (O) maya after 30 days of chronic exposure to optimal (24 °C) and heat stress (30 °C) temperatures. Bars represent mean ± SEM (n = 5 embryos per temperature). For each gene, temperatures were compared using a two-sided permutation test, followed by Benjamini–Hochberg FDR correction across genes. Statistical significance was set at q < 0.05. FC denotes fold change (heat stress/optimal) computed from means on the original scale. * indicates statistically significant differences (q < 0.05).

To further understand the transcriptional patterns observed in whole embryos of *Sepia officinalis* and *Octopus maya* under chronic heat stress, we conducted a targeted tissue-specific expression analysis. This approach aimed to determine whether the differential expression reflected a generalised systemic response or was instead modulated according to specific tissues. To this end, we quantified the expression levels of selected TRP channels in four relevant tissues, eyes, optic lobes, brain, and skin, under both control and elevated temperature conditions (**Figure 10**).

αTRPC exhibited marked tissue-specific expression patterns, with substantially higher levels in the eyes compared to other tissues examined in both species (**Figure 10**). This elevated ocular expression was not observed for the other TRP channels analysed, suggesting that αTRPC may play a particularly important role in the cephalopods eyes physiology. Furthermore, αTRPC transcript levels in the eyes were significantly upregulated under heat stress conditions in both *S. officinalis* (~2.2-fold) and *O. maya* (~3.2-fold). Among all tissues analysed, αTRPC was the most consistently heat-responsive expression patterns across both species, showing significant upregulation in the eyes as well as in whole-embryo samples.

αTRPV, which also exhibited significant upregulation at the whole-embryo level in both species, showed a different tissue-specific expression pattern compared to αTRPC. In *S. officinalis*, αTRPV expression was highest in the skin and was significantly upregulated under thermal stress exclusively in this tissue. In contrast, in *O. maya*, baseline expression levels of αTRPV were low across all tissues under control conditions, with a marked and significant upregulation observed only in the optic lobes in response to heat stress. These findings highlight species-specific differences in the tissue distribution and thermal regulation of αTRPV between *S. officinalis* and *O. maya*.

While βTRPC expression was significantly upregulated under thermal stress in whole embryos of *O. maya*, but not in *S. officinalis*, tissue-specific analyses revealed a marked increase in expression in the brain and optic lobes of both species. These findings may suggest a conserved temperature-sensitive regulation of βTRPC in neural structures, supporting its potential role in the thermal modulation of nervous system development in cephalopods.

Concerning αTRPM, which was significantly downregulated under thermal stress in the whole embryo of *S. officinalis* but not in *O. maya*, tissue-specific expression patterns revealed a similar regulatory response in both species. Under control conditions, αTRPM expression was highest in the brain and optic lobes in both species, with a significant downregulation observed under heat stress (optic lobes and brain). Additionally, a significant reduction of αTRPM transcript levels was also observed in the eye in both species and the skin of *S. officinalis*.

Overall, these tissue-specific results provide additional spatial context to the broader transcriptional responses observed at the whole-embryo level. They further highlight the usefulness of anatomically resolved gene expression profiling for identifying tissues and organs systems potentially involved in TRP-associated thermal responses during cephalopod development. In particular, the eyes emerge as key thermoresponsive tissue for αTRPC under heat stress conditions. A detailed overview of the expression profiles for all TRP genes is provided in **Supplementary Figure 11**.

## 4 Discussion

Our study expands current knowledge of TRP channel diversity and phylogenetic relationships in cephalopods. By combining genome-wide surveys with phylogenetic analyses, we identified 117 previously unannotated candidate TRP channel sequences across 13 cephalopod species and assigned them to seven major TRP families based on their phylogenetic placement, substantially expanding the currently recognised TRP repertoire in this group.

A major outcome of this work is the identification of recurrent phylogenetic groupings within several non-vertebrate TRP channel families. Specifically, our analyses consistenly recovered well-supported clades, particularly within cephalopods, corresponding to α and β groups in four major TRP channel families (TRPA, TRPV, TRPC, and TRPM), as well as an additional γ group within the TRPA and TRPV families. These phylogenetic patterns provide a framework for describing TRP channel diversity in non-vertebrates and may help clarify some of the inconsistencies associated with the application of vertebrate-based nomenclature to non-vertebrate sequences. Accordingly, we propose the use of α, β, and γ designations, where appropriate, as phylogenetically informed labels that facilitate comparative analyses across metazoan lineages. Importantly, these designations are not intended to imply direct orthology with vertebrate TRP subfamilies, but rather to provide a practical framework for describing non-vertebrate TRP diversity based on the phylogenetic relationships recovered in the present study.

Beyond taxonomic clarification, our findings reveal substantial diversity within the cephalopod TRP channel repertoire. Representatives of all seven major TRP channel families: TRPA, TRPN, TRPV, TRPC, TRPML, TRPP and TRPM, were recovered across the cephalopod species analysed. Notably, representatives of most families were recovered in Octopodiformes, Decapodiformes and Nautiloidea, suggesting that a substantial proportion of the cephalopod TRP repertoire may have been established prior to the diversification of extant cephalopod lineages. Although broader taxonomic sampling will be required to formally test this hypothesis, the observed distribution is consistent with an ancient origin of these channel families within Cephalopoda. Particularly noteworthy is the recovery of several TRP groups in *Nautilus macromphalus*, which occupies a key phylogenetic position among extant cephalopods and provides additional context for interpreting the evolutionary history of these channels.

At the same time, variation in the distribution of specific subtypes, such as the apparent restriction of αTRPA to Octopodiform species in the present dataset, together with the occurrence of multiple paralogues within certain groups (e.g., αTRPA, γTRPA and βTRPC), indicates that diversification has occurred within particular cephalopod lineages. These patterns are consistent with lineage-specific gene duplication and/or differential gene retention events, although additional genomic analyses will be required to clarify their evolutionary origins. While the functional consequences of these patterns remain unknown, they raise the possibility that diversification within some TRP families contributed to lineage-specific variation in sensory or physiological processes.

Our results not only provide a phylogenetically informed framework for the classification of non-vertebrate TRP channels but also highlight the evolutionary plasticity of the TRP gene family in cephalopods. This expanded and reclassified TRP repertoire provides a strong foundation for future functional studies aimed at elucidating the physiological roles of these channels, particularly about environmental sensing and thermal adaptation, in this ecologically and evolutionarily important group of marine invertebrates. This comprehensive identification and classification align with previous findings that underscore both the broad functional versatility and the evolutionary conservation of TRP channels across diverse taxa (Himmel and Cox, 2020; Kashio and Tominaga, 2022; Morini et al., 2022).

While the phylogenetic analyses provide insight into the evolutionary diversification of TRP channels in cephalopods, the expression analyses offer an opportunity to explore the potential functional significance of this diversity. TRP channels are widely recognised as mediators of environmental sensing and physiological homeostasis, including responses to temperature, mechanical stimuli, and osmotic changes (Jo et al., 2022; Kashio and Tominaga, 2022; Patapoutian et al., 2003; Peng et al., 2021). Consequently, variation in the expression of specific TRP channel subtypes under chronic thermal stress may provide insight into the molecular mechanisms associated with thermal responsiveness in late-stage cephalopod embryos. In this context, the comparative analysis of *Sepia officinalis* and *Octopus maya* provides an opportunity to investigate how members of the cephalopod TRP repertoire are transcriptionally regulated under elevated temperature.

Several TRP channels subtypes exhibited contrasting regulatory responses between *Sepia officinalis* and *Octopus maya*. These species occupy markedly different thermal environments, with *S. officinalis* inhabiting temperate Atlantic waters and *O. maya* occurring in the warmer Gulf of Mexico. Although the present study was not designed to directly test evolutionary adaptation, the observed differences in transcriptional responses are consistent with the possibility that these species employ distinct physiological strategies when responding to thermal stress. Given the ecological and thermal differences between these taxa, such variation may reflect species-specific regulatory responses, although additional comparative data will be required to evaluate this hypothesis. Future studies incorporating a broader range of cephalopod species occupying diverse thermal habitats will be necessary to determine whether these expression patterns represent lineage-specific responses or more general ecological trends associated with thermal adaptation.

It is important to note that the comparative framework adopted in the present study was not intended to establish direct quantitative comparisons between *Sepia officinalis* and *Octopus maya*. These species differ substantially in their developmental schedules, thermal ecology, and experimental temperature regimes. Consequently, the expression patterns reported here should be interpreted primarily in terms of common and species-specific transcriptional responses to chronic thermal stress within each species. Nevertheless, the identification of recurrent responses in phylogenetically distant cephalopods provides a useful foundation for exploring potentially conserved mechanisms of thermal responsiveness across the group.

Within this broader context, the molecular response to chronic thermal stress was not restricted to a single TRP channel subtype. Rather, several members of the TRPC, TRPV, TRPA and TRPM families exhibited significant temperature-dependent expression changes, suggesting that thermal responsiveness in cephalopods may involve the coordinated regulation of multiple TRP channel families. Consequently, the expression patterns described below should be interpreted as components of a broader molecular response to thermal stress rather than evidence for the exclusive involvement of any individual TRP subtype.

Interestingly, not all TRP channel subtypes exhibited conserved responses between species. For example, members of the TRPML and TRPP families displayed contrasting transcriptional patterns, being downregulated under thermal stress in *Sepia officinalis* but upregulated in *Octopus maya*. Although the physiological significance of these differences remains unclear, they may reflect species-specific regulatory strategies associated with adaptation to distinct thermal environments. *S. officinalis* naturally inhabits temperate waters characterized by marked seasonal temperature fluctuations, whereas *O. maya* is restricted to the comparatively warm tropical waters of the Gulf of Mexico, where elevated temperatures are a major ecological constraint (Rosa et al., 2012; Juárez et al., 2015; Caamal-Monsreal et al., 2016). Under this scenario, differential regulation of TRPML and TRPP channels may reflect lineage-specific mechanisms involved in maintaining cellular homeostasis, intracellular signalling, or developmental stability under thermal stress. However, additional physiological and functional studies will be required to determine whether these contrasting expression patterns are linked to ecological adaptation, developmental plasticity, or other species-specific regulatory processes.

From a tissue-level perspective, the observed expression patterns suggest that thermal responsiveness during cephalopod embryogenesis may involve both peripheral and central tissues. In particular, the recurrent temperature-dependent regulation of several TRP channel subtypes in the eyes and optic lobes highlights these structures as candidate tissues involved in the integration of environmental information during thermal stress, whereas the expression of TRPV channels in the skin suggests that peripheral tissues may also contribute to responses to changing thermal conditions. Although these observations do not demonstrate direct thermosensory function, they provide a biological framework for interpreting the tissue-specific expression patterns described below.

Among the TRP channel subtypes analysed, the αTRPC emerged as the most consistently and highly expressed TRP channel subtype in both whole embryos of *Sepia officinalis* and *Octopus maya* subjected to chronic thermal stress. Tissue-level profiling further revealed that its high expression and upregulation were predominantly localised to the eyes in both species. This expression pattern indicates that αTRPC is enriched in ocular tissues and suggests that this channel may be associated with molecular pathways responsive to thermal conditions within the developing visual system. However, the physiological significance of this expression pattern remains to be determined experimentally.

The marked expression of αTRPC observed in the eyes of *S. officinalis* and *O. maya* embryos aligns with previous research in a range of non-vertebrate taxa, where TRPC channels have been implicated in eye function. Notably, in *D. melanogaster*, a member of the TRPC family, annotated as TRP-gamma (corresponding to αTRPC subtype in our classification), has been extensively implicated in visual processes, mediating light-induced calcium influx in photoreceptor cells (Montell, 2005; Jörs et al., 2006). While functional studies in marine invertebrates remain limited, transcriptomic and proteomic analyses have identified enriched expression of TRPC genes in the ocular tissues of several molluscan species. For instance, TRPC transcripts have been reported in the eyes of clams (*Chlamys farreri*), where they are thought to contribute to photoreceptive function and light-modulated behaviour (Peng et al., 2021). Moreover, in echinoderms such as sea cucumber (*Apostichopus japonicus*), TRPC has been linked to light sensitivity and circadian regulation, supporting a broader role for these channels in peripheral sensory systems (Wang et al., 2023).

Collectively, these findings suggest that αTRPC may be an evolutionarily conserved component of the non-vertebrate visual system, potentially contributing to photoreception. In cephalopods, the high expression of αTRPC in ocular tissues under thermal stress raises the possibility of a dual photo- and thermo-sensory function. Although such an interpretation remains speculative, the combination of elevated ocular expression and consistent heat-induced upregulation in both *Sepia officinalis* and *Octopus maya* suggests that αTRPC may participate in pathways integrating environmental information relevant to embryonic development. This observation warrants future investigation into its possible role in ocular sensory integration. In this context, αTRPC may contribute to the processing of visual and/or temperature-related environmental cues, although its precise physiological role remains unknown. Such a dual sensory role would be particularly relevant during cephalopod embryonic development in highly variable coastal environments, where both light and temperature represent key ecological drivers.

Additional comparative insights arise from vertebrates, where TRPC channels are also linked to visual function. In mammals, TRPC1/4/5 channels, orthologous to those within the αTRPC subtype, are expressed in the retina, where they modulate calcium signalling in retinal ganglion cells and inner retinal circuits (Gilliam and Wensel, 2011). TRPC1, in particular, has been implicated in light-dependent responses and the modulation of visual signalling (Jo et al., 2022; Križaj et al., 2023), while TRPC4 and TRPC5 are found in bipolar and amacrine cells, where they influence synaptic integration and neurotransmitter release under varying light conditions (Gilliam and Wensel, 2011; Maddox et al., 2018). Complementarily, electrophysiological investigations in rainbow trout (*Oncorhynchus mykiss*) have identified corneal mechanothermal nociceptors responsive to both thermal and mechanical stimulation (Ashley et al., 2006). Collectively, these vertebrate data provide a comparative framework suggesting that αTRPC channels may fulfil sensory or modulatory functions in cephalopod ocular tissues. However, whether these functions are conserved across vertebrates and cephalopods remains to be established experimentally.

Beyond its putative involvement in sensory functions, the pronounced upregulation of αTRPC under chronic heat stress suggests a broader role in cellular stress responses. Ocular tissues are especially vulnerable to thermal challenges due to their high metabolic demands and constant exposure to the environment; in Atlantic salmon (*Salmo salar*), acute exposure to elevated temperatures (34–38 °C) induces severe lesions in gills, eyes, and brain, highlighting the susceptibility of ocular structures to heat-induced damage (Gismervik et al., 2019). In this context, αTRPC upregulation may reflect a molecular response associated with the maintenance of cellular homeostasis or sensory function under thermally challenging conditions. Consistent with this interpretation, TRPC channels have been associated with cellular stress responses in several molluscan species, including *Chlamys farreri* and *Magallana gigas*, suggesting that members of this family may participate in pathways involved in coping with environmental stressors (Peng et al., 2021; Fu et al., 2021). Although no overt morphological abnormalities were detected in *Sepia officinalis* embryos exposed to elevated temperature, chronic heat stress in *Octopus maya* has been shown to induce some developmental defects (Galindo-Torres et al., 2024). These findings underscore that thermal stress can compromise highly conserved developmental pathways in cephalopod embryos. Further comparative studies are needed to determine whether the absence of morphological alterations in *S. officinalis* reflects species-specific resilience mechanisms or simply differences in the threshold at which heat stress translates into overt phenotypic defects.

Finally, the conserved upregulation of αTRPC in representatives of both Octopodiformes and Decapodiformes is consistent with the hypothesis that this channel participates in conserved molecular responses to chronic thermal stress during embryonic development. Given the ecological importance of thermal tolerance during early developmental stages in cephalopods (Rosa et al., 2012), future functional and physiological studies will be required to determine whether αTRPC contributes directly to sensory processes, thermal responsiveness, or other aspects of cellular homeostasis.

The tissue-specific regulatory landscape under chronic thermal stress in *S. officinalis* and *O. maya* revealed that βTRPC subtype was significantly upregulated in neural tissues, specifically the optic lobes and brain, in both species, despite showing no significant modulation at the whole-embryo level in *S. officinalis*, likely due to the lack of change in the skin. This spatially confined upregulation suggests a conserved and potentially functionally relevant role for βTRPC in central thermosensory pathways and suggests that this channel may participate in pathways operating within the central nervous system. Comparable findings have been reported in other non-vertebrate taxa. In another molluscan lineage, bivalves, TRPC3-like previously annotated genes (βTRPC in our classification), have been implicated in thermal response in species such as the Pacific oyster (Fu et al., 2021). Furthermore, in vertebrate models, members of the TRPC3/6/7 family, which are phylogenetically aligned with the βTRPC clade, have been directly linked to temperature sensing and calcium signalling in neural systems. Specifically, TRPC3 and TRPC6 are known to be activated by warm temperatures and have been shown to modulate neuronal excitability and synaptic plasticity in mammalian sensory circuits (Talavera et al., 2008; Sun et al., 2020). These cross-lineage features reinforce the notion that βTRPC channel subtypes may serve conserved thermosensory or homeostatic functions in the central nervous system of marine invertebrates, particularly cephalopods.

In addition to α and βTRPC, the αTRPV channel subtype exhibited an upregulation under thermal heat stress in both *S. officinalis* and *O. maya* whole embryos. In *S. officinalis*, αTRPV expression was predominantly peripheral, in the skin, and upregulated by temperature, which may be consistent with a role in peripheral thermal responsiveness. Conversely, *O. maya* exhibited a more even distribution of αTRPV expression, including strong upregulation in the optic lobes, suggesting that this channel may also participate in neural responses to elevated temperature. Members of the TRPV family are well-recognised mediators of thermosensation across both vertebrate and invertebrate taxa. Early evidence for the involvement of TRPV channels in non-vertebrate sensory responses to noxious environmental stimuli was provided by studies in *Caenorhabditis elegans*, where mutations affecting the TRPV channel OSM-9 resulted in defective thermal avoidance behaviour, highlighting the importance of TRPV-mediated signalling in nociceptive and thermally relevant sensory pathways (Wittenburg and Baumeister, 1999). Similarly, studies in molluscs have further supported the role of TRPV channels in environmental sensing and thermal responsiveness. In bivalves, acute heat exposure increases TRPV expression in gill and mantle tissues, coordinating calcium‐dependent signalling cascades and cytoprotective heat‐shock responses (Fu et al., 2021), while in gastropods, TRPV activation within the pedal and cephalic ganglia links peripheral temperature detection to central neural circuits governing behavioural thermoregulation (Venkatachalam and Montell, 2007; Paschapur et al., 2025).

Comparable thermosensory functions have been well documented for some TRPV in vertebrates. These channels are highly expressed in both peripheral sensory neurons and central brain regions involved in thermoregulation. TRPV1 is activated by noxious heat and plays a pivotal role in pain perception and thermally evoked reflexes, while TRPV4 contributes to the detection of warm temperatures, especially within the hypothalamus, where it modulates systemic thermoregulatory output (Caterina et al., 1997; Tan and Knight, 2018; Fu et al., 2021). Moreover, in mammals, TRPV1 and TRPV4 are expressed in skin keratinocytes and dorsal root ganglia, where they transduce thermal cues into neural signals that are relayed to the spinal cord and brainstem for behavioural and autonomic regulation of body temperature (Benham et al., 2002; Vriens et al., 2004). Similar to their function in vertebrates, TRPV channels in cephalopods may act as integrators of thermal information across peripheral and central pathways. However, direct evidence for such functions in cephalopods is currently lacking.

In contrast to αTRPC and αTRPV, αTRPM exhibited a tendency towards downregulation under chronic thermal stress in both *S. officinalis* and *O. maya*, particularly in neural tissues such as the brain and optic lobes. Although the physiological significance of this response remains unclear, these results raise the possibility that αTRPM participates in neural processes that are preferentially maintained under non-stressful thermal conditions. Comparable temperature-dependent modulation of TRPM channels has also been documented in other molluscs. In the scallop *C. farreri*, expression levels of TRPM3 (corresponding to the αTRPM subtype in our classification) were found to decrease at lower temperatures (e.g., 20 °C) compared to higher ones (e.g., 27 °C) across several tissues (Peng et al., 2021). These findings support the notion of temperature-dependent regulation within this TRP family. However, the upregulation by high temperature in this bivalve contrasts with the downregulation of αTRPM observed in cephalopods in our study.

Interestingly, in vertebrate models, particularly for members of the αTRPM subtype, which includes TRPM 1/3/6/7, the downregulation of specific TRPM channels has been reported under sustained heat exposure, particularly within the central nervous system. For instance, studies in rodents (*M. musculus*) and zebrafish (*D. rerio*) have reported heat-induced suppression of TRPM3 and TRPM7 in brain regions such as the hypothalamus and spinal cord, correlating with impaired calcium signalling and altered neural excitability during thermal stress (Tan and McNaughton, 2016; Tan and Knight, 2018; Turlova et al., 2023; Li et al., 2024). These observations indicate that temperature-dependent regulation of TRPM channels is not restricted to cephalopods and may represent a more widespread feature of this channel family. Functional assays and experiments involving both acute and chronic thermal stress will be essential to clarify the specific roles of TRPM channels in modulating neural responses to temperature fluctuations.

An important consideration is that the expression patterns reported here were measured following prolonged thermal exposure. Consequently, they are likely to reflect chronic physiological responses and developmental acclimation processes rather than immediate thermosensory activation. Future studies comparing acute and chronic thermal challenges will be necessary to further resolve these complementary aspects of TRP channel function.

## 5 Conclusions

In summary, this study provides new insights into TRP channel diversity and thermal regulation in cephalopods. Through genome-wide identification and phylogenetic analyses, we expanded the currently recognised cephalopod TRP repertoire by identifying numerous previously unannotated candidate TRP sequences and assigning them to seven major TRP channel families. In addition, the recovery of recurrent α, β and γ clades within several TRP families provides a phylogenetically informed framework for describing TRP channel diversity in cephalopods and other non-vertebrate taxa.

Collectively, our findings underscore the large number of TRP types and subtypes and the evolutionary plasticity of the TRP channel family in this group, while also highlighting the broad conservation of a core TRP repertoire across distinct cephalopod lineages. These results establish a molecular framework for investigating cephalopod responses to thermal fluctuations in an increasingly warming ocean.

The observed expression patterns, combining both conserved and species-specific regulatory signatures, identify αTRPC as one of the most consistently responsive TRP channel subtypes across species and tissues examined in this study. The elevated ocular expression and conserved upregulation of αTRPC in representatives of both Octopodiformes and Decapodiformes are consistent with the possibility that this channel participates in molecular pathways responsive to thermal conditions during embryonic development. However, the precise physiological functions of αTRPC, as well as those of other thermally responsive TRP channels, remain to be determined.

To further elucidate the roles of these channels in thermosensation and thermoregulation, future research should adopt integrative approaches encompassing electrophysiological recordings, behavioural assays, and further experiments on key environmental variables, particularly temperature and light, across different exposure regimes. Future studies integrating both acute and chronic thermal challenges will also be important for clarifying the respective contributions of thermosensory signalling and longer-term developmental acclimation processes. Such investigations would clarify the functional significance of individual TRP channels under thermal stress and contribute to a broader understanding of the mechanisms underlying thermal adaptation in cephalopods. Such knowledge will be important for understanding how cephalopods perceive and respond to environmental change and may ultimately improve predictions of their resilience to ongoing ocean warming.

## Data Availability

The datasets presented in this study can be found in online repositories. The names of the repository/repositories and accession number(s) can be found in the article/**Supplementary Material**.
