# Supplementary material for: Decoding thermal adaptation: genome-wide identification of TRP channel repertoire in cephalopods and gene expression responses to chronic thermal exposure in Sepia officinalis and Octopus maya embryos
Source: Front Physiol. 2026 Jul 13;17:1851308. doi: 10.3389/fphys.2026.1851308 (PMC13402157; doi:10.3389/fphys.2026.1851308)
Supplement: Supplementary file 1 [file DataSheet1.docx]

**Decoding Thermal Adaptation: Genome-Wide Identification of TRP Channel Repertoire in Cephalopods and Gene Expression Responses to Chronic Thermal Exposure in *Sepia officinalis* and *Octopus maya* embryos**

Luis Molina-Carrillo^1^*, Marina Morini^1^, Sylvie Dufour^1^, Pavel Galindo-Torres^2^, Sadot Ramos-Rodriguez^2^, Carlos Rosas^3^, Claudia Caamal-Monsreal^3^, Clara E. Galindo-Sánchez^2^, Yann Bassaglia^1,5^ and Laure Bonnaud-Ponticelli^1^*

^1^Laboratoire de Biologie des Organismes et des Écosystèmes Aquatiques-BOREA. Muséum national d’Histoire naturelle (MNHN), SU, CNRS, IRD, UA, F-75005 Paris, France

^2^Centro de Investigación Científica y de Educación Superior de Ensenada (CICESE). Carretera Tijuana-Ensenada 6 No. 3918, Zona Playitas, Ensenada, Baja California, CP 22860. México.

^3^Unidad Multidisciplinaria de Docencia e Investigación (UMDI), Facultad de Ciencias, Universidad Nacional Autonóma de México (UNAM), Puerto DE Abrigo s/n, Sisal, Hunucma, Yucatán CP97355, México.

^4^Station Biologique de Roscoff, CNRS-Sorbonne Université, Roscoff 29682, France.

^5^Université Paris Est Créteil-Val de Marne (UPEC), France.

***** Corresponding Author
[luis-miguel.molina-carrillo@mnhn.fr](mailto:luis-miguel.molina-carrillo@mnhn.fr)

laure.bonnaud@mnhn.fr

**Appendix A. Supplementary data**

**Supplemental Table S1.**

The accession numbers for the candidates' TRP proteins are attached in Excel format.

**Supplemental Table S2.**

**Table S2**: Primers for quantitative expression analyses.

| ***Sepia officinalis*** | | | |
| --- | --- | --- | --- |
| GeneID | Sequences (5′ → 3′) | | Size (bp) |
| So_βTRPA | F:ATGGACCGTTGTACCACACC  R:ACCGGAATCATCTTCAGGGC | | 102 |
| So_αTRPC | F:TGAAGAATGGAAGTTTGCCCG  R:AATGGTGGTGGTACAGTGGC | | 75 |
| So_βTRPC | F:AGTTCGTTGCACATCCAAGC  R:CGGGTGTACCAGTTTGACCT | | 91 |
| So_αTRPM | F:GCGAATGTTCCTATGGTGCG  R:TCACGAGAGTTGGCGTTGTT | | 136 |
| So_βTRPM | F:CAATGGCCCGCTCTTCAAAAA  R:GGAAGGAAGGAATGGAAGGCT | | 138 |
| So_αTRPV | F:AACTGCTGGACGAGAAGTGG  R:TGAAGCCCAGAAGTTGGGTC | | 135 |
| So_βTRPV | F:GCCAAAGGATATTCGCCCCA  R:TCCAGGACTGACAACTGGGA | | 92 |
| So_TRPML | F:CGTCGCATACGATCCTCCAT  R:CCATGGTCGGCACCATCAA | | 130 |
| So_TRPP | F:GTGGGACTTTGGTGATGGCT  R:ATGACCCCATGGAGAGTGGA | | 153 |
| ***Octopus maya*** | | | |
| GeneID | | Sequences (5′ → 3′) | Size (bp) |
| Om_βTRPA | | F:AGCATGGATTGATTTGGTGCTC  R:ACCCAAATGCAAATGCAACAA | 140 |
| Om_αTRPC | | F:GCAGAAGCCTTGTTTGCTGG  R:ATCTGCCACCATTCGACCAA | 117 |
| Om_βTRPC | | F:GTCGCCTTACCCCAAAGACT  R:CTGCAGCGTACACTTGCTTC | 119 |
| Om_αTRPM | | F:CCAGCGAGCTTGGATTGAGA  R:GTCAGCAGCAATTTCCCGTG | 139 |
| Om_βTRPM | | F:GGATCATGGCCTGCAGAGTA  R:CCAAAAGTGTCTGAGGTACCATT | 119 |
| Om_αTRPV | | F:ACAGCATCTATTGCACCTCTATATT  R:TACTAGTTTTCGCCTTCTTACACC | 100 |
| Om_βTRPV | | F:AACCAAGCTCACTACTCCACG  R:CGGACTGGTATGTGTTTCCCA | 115 |
| Om_TRPML | | F:CCTCAAGAGAGCTGCACCAA  R:TGTGGTGCTCAAATGACGGA | 130 |
| Om_TRPP | | F:TCCAGGAATGGGCCTGTAGA  R:GGATGGCATGGACGGTTTAC | 113 |

**Supplemental Table S3.**

**Table S3** dMIQE checklist for dPCR experiments.

| **ITEM TO CHECK** | **PROVIDED** | **COMMENT** |
| --- | --- | --- |
| **Column1** | **Y/N** | **Column2** |
| **1. SPECIMEN** |  |  |
| Detailed description of specimen type and numbers | **Y** | METHODS |
| Sampling procedure (including time to storage) | **Y** | METHODS |
| Sample aliquotation, storage conditions and duration | **Y** | METHODS |
| **2. NUCLEIC ACID EXTRACTION** |  |  |
| Description of extraction method including amount of sample processed | **Y** | METHODS |
| Volume of solvent used to elute/resuspend extract | **Y** | METHODS |
| Number of extraction replicates | **Y** | METHODS |
| Extraction blanks included? | **Y** | METHODS |
| **3. NUCLEIC ACID ASSESSMENT AND STORAGE** |  |  |
| Method to evaluate quality of nucleic acids | **Y** | METHODS |
| Method to evaluate quantity of nucleic acids (including molecular weight and calculations when using mass) | **Y** | METHODS |
| Storage conditions: temperature, concentration, duration, buffer, aliquots | **Y** | METHODS |
| Clear description of dilution steps used to prepare working DNA solution | **N** | N/A |
| **4. NUCLEIC ACID MODIFICATION** |  |  |
| Template modification (digestion, sonication, pre-amplification, bisulphite etc.) | **N** | N/A |
| Details of repurification following modification if performed | **N** | N/A |
| **5. REVERSE TRANSCRIPTION** |  |  |
| cDNA priming method and concentration | **Y** | METHODS |
| One or two step protocol (include reaction details for two step) | **Y** | METHODS |
| Amount of RNA added per reaction | **Y** | METHODS |
| Detailed reaction components and conditions | **Y** | METHODS |
| Estimated copies measured with and without addition of RT* | **N** | ND |
| Manufacturer of reagents used with catalogue and lot numbers | **Y** | METHODS |
| Storage of cDNA: temperature, concentration, duration, buffer and aliquots | **Y** | METHODS |
| **6. dPCR OLIGONUCLEOTIDES DESIGN AND TARGET INFORMATION** |  |  |
| Sequence accession number or official gene symbol | **Y** | METHODS |
| Method (software) used for design and *in silico* verification | **Y** | METHODS |
| Location of amplicon | **N** | N/A |
| Amplicon length | **Y** | METHODS |
| Primer and probe sequences (or amplicon context sequence)** | **Y** | METHODS |
| Location and identity of any modifications | **N** | ND |
| Manufacturer of oligonucleotides | **Y** | METHODS |
| **7. dPCR PROTOCOL** |  |  |
| Manufacturer of dPCR instrument and instrument model | **Y** | Table S4 |
| Buffer/kit manufacturer with catalogue and lot number | **Y** | Table S4 |
| Primer and probe concentration | **Y** | METHODS |
| Pre-reaction volume and composition (incl. amount of template and if restriction enzyme added) | **N** | ND |
| Template treatment (initial heating or chemical denaturation) | **N** | ND |
| Polymerase identity and concentration, Mg++ and dNTP concentrations*** | **Y** | METHODS |
| Complete thermocycling parameters | **Y** | METHODS |
| **8. ASSAY VALIDATION** |  |  |
| Details of optimisation performed | **Y** | Table S5/S6 |
| Analytical specificity (vs. related sequences) and limit of blank (LOB) | **N** | ND |
| Analytical sensitivity/LoD and how this was evaluated | **Y** | Figure S1/S2 |
| Testing for inhibitors (from biological matrix/extraction) | **N** | ND |
| **9. DATA ANALYSIS** |  |  |
| Description of dPCR experimental design | **Y** | METHODS |
| Comprehensive details negative and positive of controls (whether applied for QC or for estimation of error) | **Y** | Figure S3 and Table S5/S6 |
| Partition classification method (thresholding) | **Y** | METHODS |
| Examples of positive and negative experimental results (including fluorescence plots in supplemental material) | **N** | N/A |
| Description of technical replication | **Y** | METHODS |
| Repeatability (intra-experiment variation) | **Y** | Figure S3 and Table S5/S6 |
| Reproducibility (inter-experiment/user/lab etc. variation ) | **N** | N/A |
| Number of partitions measured (average and standard deviation ) | **Y** | Figure S3 and Table S5/S6 |
| Partition volume | **Y** | METHODS |
| Copies per partition (λ or equivalent ) (average and standard deviation) | **Y** | Table S5/S6 |
| dPCR analysis program (source, version) | **Y** | Table S4 |
| Description of normalisation method | **Y** | N/A |
| Statistical methods used for analysis | **Y** | METHODS |
| Data transparency | raw data available on request: | Y |
|  |  |  |
| **Table S1.** dMIQE2020 checklist for authors, reviewers and editors. Authors should fill detail whether information is provided. Where ‘yes’ is selected use comment box to detail location of information or to include the information. Where ‘no’ is selected use comment box to outline rationale for omission. Sections 4 and 5 may not apply depending on experiment. | | |
|  |  |  |
| * Assessing the absence of DNA using a no RT assay (or where RT has been inactivated) is essential when first extracting RNA. Once the sample has been validated as DNA-free, inclusion of a no-RT control is desirable, but no longer essential. | | |
|  |  |  |
| ** Disclosure of the primer and probe sequence is highly desirable and strongly encouraged. However, since not all commercial pre-designed assay vendors provide this information when it is not available assay context sequences must be submitted (Bustin et al. Primer sequence disclosure: A clarification of the miqe guidelines. Clin Chem 2011;57:919-21.) | | |
|  |  |  |
| *** Details of reaction components is highly desirable, however not always possible for commercial disclosure reasons. Inclusion of catalogue number is essential where component reagent details are not available. | | |

**Supplemental Table S4. dPCR protocol**

| **dPCR platform** | **QIAcuity One Digital PCR System**  **(Qiagen)** |
| --- | --- |
| QIAcuity 8.5K 96-well Nanoplates (Cat No. 250021) | |
| Prepared reaction volume (µL) | 12 |
| Partition volume | 0.34 nL |
| Theorical maximum partition number | 8500 |
| Minimun number of partition accepted to pass QC | ≥8100 |
| Mastermix | QIAcuity EG PCR master mix (Cat No. 250113) |
| Analysis sotfware | QIAcuitySoftwareSuite_2.2.5 |

**Table S5**. dPCR assay validation and data analysis of *S. officinalis* primers

| **Target** | **Treatment (°C)** | **Dilution factor** | **Conc[copies/µL]^a^** | **Mean** | **SD** | **Partitions (Valid)^b^** | **Partitions (Positive)^c^** |
| --- | --- | --- | --- | --- | --- | --- | --- |
| βTRPA | 16 | *0.2* | 128,4 |  |  | 8248 | 319 |
| βTRPA | 16 |  | 129,4 | 128,9 | 0,7 | 8300 | 333 |
| βTRPA | 16 | *0.15* | 76,78 |  |  | 8305 | 193 |
| βTRPA | 16 |  | 74,83 | 75,8 | 1,4 | 8274 | 192 |
| βTRPA | 16 | *0.1* | 42,73 |  |  | 8300 | 109 |
| βTRPA | 16 |  | 34,75 | 38,7 | 5,6 | 8269 | 90 |
| βTRPA | 16 | *0.05* | 6,922 |  |  | 8235 | 18 |
| βTRPA | 16 |  | 4,341 | 5,6 | 1,8 | 7123 | 10 |
| NTC |  | *NTC^d^* | 0 |  |  | 8268 | 0 |
| NTC |  |  | 0 | 0,0 | 0,0 | 8268 | 0 |
| βTRPA | 22 | *0.2* | 71,77 |  |  | 8246 | 190 |
| βTRPA | 22 |  | 93,79 | 82,8 | 15,6 | 8290 | 249 |
| βTRPA | 22 | *0.15* | 27,85 |  |  | 8289 | 72 |
| βTRPA | 22 |  | 33,54 | 30,7 | 4,0 | 8307 | 89 |
| βTRPA | 22 | *0.1* | 17,9 |  |  | 8294 | 46 |
| βTRPA | 22 |  | 14,37 | 16,1 | 2,5 | 8298 | 38 |
| βTRPA | 22 | *0.05* | 1,945 |  |  | 8280 | 5 |
| βTRPA | 22 |  | 3,791 | 2,9 | 1,3 | 8289 | 10 |
| NTC |  | *NTC^d^* | 0 |  |  | 8268 | 0 |
| NTC |  |  | 0 | 0,0 | 0,0 | 8268 | 0 |
|  |  |  |  |  |  |  |  |
| αTRPC | 16 | *0.2* | 164,4 |  |  | 8251 | 424 |
| αTRPC | 16 |  | 151,9 | 158,2 | 8,8 | 8290 | 394 |
| αTRPC | 16 | *0.15* | 75,38 |  |  | 8260 | 193 |
| αTRPC | 16 |  | 76,88 | 76,1 | 1,1 | 8249 | 195 |
| αTRPC | 16 | *0.1* | 28,24 |  |  | 8213 | 71 |
| αTRPC | 16 |  | 30,58 | 29,4 | 1,7 | 8218 | 77 |
| αTRPC | 16 | *0.05* | 7,048 |  |  | 8232 | 18 |
| αTRPC | 16 |  | 5,526 | 6,3 | 1,1 | 8287 | 14 |
| αTRPC | 16 | *NTC^d^* | 0 |  |  | 8282 | 0 |
| αTRPC | 16 |  | 0,396 | 0,2 | 0,3 | 8250 | 1 |
|  |  |  |  |  |  |  |  |
| αTRPC | 22 | *0.2* | 1943,8 |  |  | 7921 | 3511 |
| αTRPC | 22 |  | 1693 | 1818,4 | 177,3 | 8280 | 3333 |
| αTRPC | 22 | *0.15* | 886,1 |  |  | 8186 | 1935 |
| αTRPC | 22 |  | 894,6 | 890,4 | 6,0 | 8189 | 1925 |
| αTRPC | 22 | *0.1* | 366,8 |  |  | 8201 | 864 |
| αTRPC | 22 |  | 380,5 | 373,7 | 9,7 | 8253 | 896 |
| αTRPC | 22 | *0.05* | 65,6 |  |  | 8287 | 167 |
| αTRPC | 22 |  | 66,1 | 65,9 | 0,4 | 8282 | 165 |
| αTRPC | 22 | *NTC^d^* | 0 |  |  | 8258 | 0 |
| αTRPC | 22 |  | 0 | 0,0 | 0,0 | 8218 | 0 |
|  |  |  |  |  |  |  |  |
| βTRPC | 16 | *0.2* | 7,634 |  |  | 8292 | 20 |
| βTRPC | 16 |  | 6,904 | 7,3 | 0,5 | 8288 | 18 |
| βTRPC | 16 | *0.15* | 5,488 |  |  | 8233 | 14 |
| βTRPC | 16 |  | 5,435 | 5,5 | 0,0 | 8220 | 14 |
| βTRPC | 16 | *0.1* | 1,972 |  |  | 8225 | 5 |
| βTRPC | 16 |  | 2,776 | 2,4 | 0,6 | 8228 | 7 |
| βTRPC | 16 | *0.05* | 0 |  |  | 8287 | 0 |
| βTRPC | 16 |  | 0 | 0,0 | 0,0 | 8268 | 0 |
| βTRPC | 16 | *NTC^d^* | 0 |  |  | 8214 | 0 |
| βTRPC | 16 |  | 0 | 0,0 | 0,0 | 8292 | 0 |
|  |  |  |  |  |  |  |  |
| βTRPC | 22 | *0.2* | 2,019 |  |  | 8224 | 5 |
| βTRPC | 22 |  | 5,217 | 3,6 | 2,3 | 8270 | 13 |
| βTRPC | 22 | *0.15* | 2,044 |  |  | 8261 | 5 |
| βTRPC | 22 |  | 1,617 | 1,8 | 0,3 | 8279 | 4 |
| βTRPC | 22 | *0.1* | 0,815 |  |  | 8213 | 2 |
| βTRPC | 22 |  | 0,4 | 0,6 | 0,3 | 8266 | 1 |
| βTRPC | 22 | *0.05* | 0,4 |  |  | 8267 | 1 |
| βTRPC | 22 |  | 0,796 | 0,6 | 0,3 | 8279 | 2 |
| βTRPC | 22 | *NTC^d^* | 0,401 |  |  | 8258 | 1 |
| βTRPC | 22 |  | 0 | 0,2 | 0,3 | 8209 | 0 |
|  |  |  |  |  |  |  |  |
| αTRPM | 16 | *0.2* | 51,5 |  |  | 8287 | 135 |
| αTRPM | 16 |  | 47,04 | 49,3 | 3,2 | 8251 | 126 |
| αTRPM | 16 | *0.15* | 28,8 |  |  | 8301 | 74 |
| αTRPM | 16 |  | 26,25 | 27,5 | 1,8 | 8267 | 70 |
| αTRPM | 16 | *0.1* | 10,16 |  |  | 8281 | 26 |
| αTRPM | 16 |  | 9,518 | 9,8 | 0,5 | 8264 | 25 |
| αTRPM | 16 | *0.05* | 2,351 |  |  | 8253 | 6 |
| αTRPM | 16 |  | 0,379 | 1,4 | 1,4 | 8232 | 1 |
| αTRPM | 16 | *NTC^d^* | 0 |  |  | 8267 | 0 |
| αTRPM | 16 |  | 0 | 0,0 | 0,0 | 8267 | 0 |
|  |  |  |  |  |  |  |  |
| αTRPM | 22 | *0.2* | 37,94 |  |  | 8282 | 95 |
| αTRPM | 22 |  | 35,57 | 36,8 | 1,7 | 8244 | 93 |
| αTRPM | 22 | *0.15* | 18,12 |  |  | 8248 | 45 |
| αTRPM | 22 |  | 19,04 | 18,6 | 0,7 | 8258 | 50 |
| αTRPM | 22 | *0.1* | 9,049 |  |  | 8277 | 23 |
| αTRPM | 22 |  | 9,848 | 9,4 | 0,6 | 8252 | 26 |
| αTRPM | 22 | *0.05* | 1,179 |  |  | 8201 | 3 |
| αTRPM | 22 |  | 0,754 | 1,0 | 0,3 | 8253 | 2 |
| αTRPM | 22 | *NTC^d^* | 0 |  |  | 8225 | 0 |
| αTRPM | 22 |  | 0 | 0,0 | 0,0 | 8251 | 0 |
|  |  |  |  |  |  |  |  |
| βTRPM | 16 | *0.2* | 171,1 |  |  | 8266 | 419 |
| βTRPM | 16 |  | 113 | 142,1 | 41,1 | 8222 | 291 |
| βTRPM | 16 | *0.15* | 79,63 |  |  | 8291 | 197 |
| βTRPM | 16 |  | 81,24 | 80,4 | 1,1 | 8250 | 211 |
| βTRPM | 16 | *0.1* | 39,43 |  |  | 8298 | 100 |
| βTRPM | 16 |  | 49,55 | 44,5 | 7,2 | 8253 | 130 |
| βTRPM | 16 | *0.05* | 6,223 |  |  | 8290 | 16 |
| βTRPM | 16 |  | 5,735 | 6,0 | 0,3 | 8140 | 15 |
| βTRPM | 16 | *NTC^d^* | 0 |  |  | 8256 | 0 |
| βTRPM | 16 |  | 0 | 0,0 | 0,0 | 8231 | 0 |
|  |  |  |  |  |  |  |  |
| βTRPM | 22 | *0.2* | 60,91 |  |  | 8265 | 159 |
| βTRPM | 22 |  | 65,5 | 63,2 | 3,2 | 8255 | 175 |
| βTRPM | 22 | *0.15* | 27,4 |  |  | 8253 | 70 |
| βTRPM | 22 |  | 27,12 | 27,3 | 0,2 | 8231 | 72 |
| βTRPM | 22 | *0.1* | 18,03 |  |  | 8266 | 46 |
| βTRPM | 22 |  | 14,1 | 16,1 | 2,8 | 8262 | 37 |
| βTRPM | 22 | *0.05* | 0,392 |  |  | 8259 | 1 |
| βTRPM | 22 |  | 0,377 | 0,4 | 0,0 | 8266 | 1 |
| βTRPM | 22 | *NTC^d^* | 0 |  |  | 8253 | 0 |
| βTRPM | 22 |  | 0,001 | 0,0 | 0,0 | 8253 | 0 |
|  |  |  |  |  |  |  |  |
| αTRPV | 16 | *0.2* | 3,179 |  |  | 8278 | 8 |
| αTRPV | 16 |  | 3,803 | 3,5 | 0,4 | 8250 | 10 |
| αTRPV | 16 | *0.15* | 2,807 |  |  | 8262 | 7 |
| αTRPV | 16 |  | 1,137 | 2,0 | 1,2 | 8273 | 3 |
| αTRPV | 16 | *0.1* | 0,393 |  |  | 8276 | 1 |
| αTRPV | 16 |  | 0,379 | 0,4 | 0,0 | 8244 | 1 |
| αTRPV | 16 | *0.05* | 0 |  |  | 8255 | 0 |
| αTRPV | 16 |  | 0 | 0,0 | 0,0 | 8211 | 0 |
| αTRPV | 16 | *NTC^d^* | 0,382 |  |  | 8281 | 0 |
| αTRPV | 16 |  | 0,382 | 0,4 | 0,0 | 8246 | 1 |
|  |  |  |  |  |  |  |  |
| αTRPV | 22 | *0.2* | 5,893 |  |  | 8284 | 15 |
| αTRPV | 22 |  | 6,202 | 6,0 | 0,2 | 8233 | 16 |
| αTRPV | 22 | *0.15* | 2,626 |  |  | 8301 | 6 |
| αTRPV | 22 |  | 2,988 | 2,8 | 0,3 | 8267 | 8 |
| αTRPV | 22 | *0.1* | 0,782 |  |  | 8260 | 2 |
| αTRPV | 22 |  | 1,522 | 1,2 | 0,5 | 8260 | 4 |
| αTRPV | 22 | *0.05* | 0 |  |  | 8289 | 0 |
| αTRPV | 22 |  | 0 | 0,0 | 0,0 | 8257 | 0 |
| αTRPV | 22 | *NTC^d^* | 0,397 |  |  | 8289 | 0 |
| αTRPV | 22 |  | 0,379 | 0,4 | 0,0 | 8257 | 0 |
|  |  |  |  |  |  |  |  |
| TRPP | 16 | *0.2* | 57,46 |  |  | 8280 | 152 |
| TRPP | 16 |  | 48,32 | 52,9 | 6,5 | 8271 | 123 |
| TRPP | 16 | *0.15* | 17,3 |  |  | 8272 | 45 |
| TRPP | 16 |  | 21,05 | 19,2 | 2,7 | 8279 | 54 |
| TRPP | 16 | *0.1* | 9,801 |  |  | 8237 | 26 |
| TRPP | 16 |  | 10,69 | 10,2 | 0,6 | 8268 | 27 |
| TRPP | 16 | *0.05* | 2,255 |  |  | 8272 | 6 |
| TRPP | 16 |  | 4,269 | 3,3 | 1,4 | 8270 | 11 |
| TRPP | 16 | *NTC^d^* | 0,377 |  |  | 8271 | 1 |
| TRPP | 16 |  | 0 | 0,2 | 0,3 | 8263 | 0 |
|  |  |  |  |  |  |  |  |
| TRPP | 22 | *0.2* | 23,07 |  |  | 8218 | 62 |
| TRPP | 22 |  | 21,54 | 22,3 | 1,1 | 8285 | 57 |
| TRPP | 22 | *0.15* | 9,45 |  |  | 8232 | 25 |
| TRPP | 22 |  | 10,23 | 9,8 | 0,6 | 8279 | 27 |
| TRPP | 22 | *0.1* | 2,638 |  |  | 8272 | 7 |
| TRPP | 22 |  | 3,895 | 3,3 | 0,9 | 8207 | 10 |
| TRPP | 22 | *0.05* | 0,378 |  |  | 8259 | 1 |
| TRPP | 22 |  | 1,17 | 0,8 | 0,6 | 8251 | 3 |
| TRPP | 22 | *NTC^d^* | 0 |  |  | 8268 | 0 |
| TRPP | 22 |  | 0 | 0,0 | 0,0 | 8279 | 0 |
|  |  |  |  |  |  |  |  |
| TRPML | 16 | *0.2* | 137,6 |  |  | 8271 | 336 |
| TRPML | 16 |  | 108,2 | 122,9 | 20,8 | 8277 | 268 |
| TRPML | 16 | *0.15* | 61,13 |  |  | 8246 | 152 |
| TRPML | 16 |  | 48,01 | 54,6 | 9,3 | 8264 | 118 |
| TRPML | 16 | *0.1* | 25,22 |  |  | 8261 | 63 |
| TRPML | 16 |  | 26,49 | 25,9 | 0,9 | 8282 | 66 |
| TRPML | 16 | *0.05* | 6,618 |  |  | 8286 | 17 |
| TRPML | 16 |  | 7,557 | 7,1 | 0,7 | 8267 | 19 |
| TRPML | 16 | *NTC^d^* | 0 |  |  | 8273 | 0 |
| TRPML | 16 |  | 0 | 0,0 | 0,0 | 8293 | 0 |
|  |  |  |  |  |  |  |  |
| TRPML | 22 | *0.2* | 52,42 |  |  | 8276 | 138 |
| TRPML | 22 |  | 47,33 | 49,9 | 3,6 | 8235 | 124 |
| TRPML | 22 | *0.15* | 28,25 |  |  | 8277 | 73 |
| TRPML | 22 |  | 22,63 | 25,4 | 4,0 | 8266 | 58 |
| TRPML | 22 | *0.1* | 13,76 |  |  | 8293 | 35 |
| TRPML | 22 |  | 12,95 | 13,4 | 0,6 | 8292 | 33 |
| TRPML | 22 | *0.05* | 1,559 |  |  | 8263 | 4 |
| TRPML | 22 |  | 2,762 | 2,2 | 0,9 | 8286 | 7 |
| TRPML | 22 | *NTC^d^* | 0 |  |  | 8245 | 0 |
| TRPML | 22 |  | 0 | 0,0 | 0,0 | 8266 | 0 |

**^a^** Number of target gene (copies/µL) in whole embryos dilution series measured by dPCR and estimated by a Poisson distribution to the ratio of positive to negative partitions.

**^b^** Total number of partitions in dPCR reaction.

**^c^** Number of partitions showing a positive signal for each target gene.

**^d^** NTC= no template control.

**Table S6**. dPCR assay validation and data analysis of *O. maya* primers

| **Target** | **Treatment (°C)** | **Dilution factor** | **Conc[copies/µL]^a^** | **Mean** | **SD** | **Partitions (Valid)^b^** | **Partitions (Positive)^c^** |
| --- | --- | --- | --- | --- | --- | --- | --- |
| βTRPA | 24 | *0.2* | 10,07 |  |  | 8262 | 28 |
| βTRPA | 24 |  | 8,016 | 9,0 | 1,5 | 8294 | 20 |
| βTRPA | 24 | *0.15* | 4,491 |  |  | 8274 | 11 |
| βTRPA | 24 |  | 4,853 | 4,7 | 0,3 | 8277 | 12 |
| βTRPA | 24 | *0.1* | 1,615 |  |  | 8288 | 4 |
| βTRPA | 24 |  | 1,995 | 1,8 | 0,3 | 8296 | 5 |
| βTRPA | 24 | *0.05* | 0 |  |  | 8274 | 0 |
| βTRPA | 24 |  | 0,397 | 0,2 | 0,3 | 8300 | 1 |
| NTC | 24 | *NTC^d^* | 0 |  |  | 8274 | 0 |
| NTC | 24 |  | 0 | 0,0 | 0,0 | 8274 | 0 |
| βTRPA |  | *0.2* |  |  |  |  |  |
| βTRPA | 30 |  | 17,64 |  |  | 8267 | 46 |
| βTRPA | 30 | *0.15* | 15,78 | 16,7 | 1,3 | 8265 | 44 |
| βTRPA | 30 |  | 7,111 |  |  | 8269 | 19 |
| βTRPA | 30 | *0.1* | 7,698 | 7,4 | 0,4 | 8269 | 21 |
| βTRPA | 30 |  | 1,173 |  |  | 8260 | 5 |
| βTRPA | 30 | *0.05* | 2,756 | 2,0 | 1,1 | 8289 | 7 |
| βTRPA | 30 |  | 0,392 |  |  | 8271 | 1 |
| NTC | 30 | *NTC^d^* | 0,402 | 0,4 | 0,0 | 8271 | 1 |
| NTC | 30 |  | 0 |  |  | 8275 | 0 |
|  |  |  |  |  |  |  |  |
| αTRPC | 24 | *0.2* | 42,4 |  |  | 8248 | 112 |
| αTRPC | 24 |  | 37,67 | 40,0 | 3,3 | 8265 | 96,0 |
| αTRPC | 24 | *0.15* | 19,61 |  |  | 8271 | 51 |
| αTRPC | 24 |  | 22,29 | 21,0 | 1,9 | 8257 | 57 |
| αTRPC | 24 | *0.1* | 13,14 |  |  | 8275 | 35 |
| αTRPC | 24 |  | 9,12 | 11,1 | 2,8 | 8250 | 23,0 |
| αTRPC | 24 | *0.05* | 1,509 |  |  | 8241 | 4 |
| αTRPC | 24 |  | 1,162 | 1,3 | 0,2 | 8284 | 3 |
| αTRPC | 24 | *NTC^d^* | 0 |  |  | 8204 | 0 |
| αTRPC | 24 |  | 0,389 | 0,2 | 0,3 | 8278,0 | 1,0 |
|  |  |  |  |  |  |  |  |
| αTRPC | 30 | *0.2* | 97,63 |  |  | 8242 | 260 |
| αTRPC | 30 |  | 104,2 | 100,9 | 4,6 | 8281 | 272 |
| αTRPC | 30 | *0.15* | 40,57 |  |  | 8249 | 107 |
| αTRPC | 30 |  | 36,47 | 38,5 | 2,9 | 8293 | 96 |
| αTRPC | 30 | *0.1* | 21,91 |  |  | 8279 | 58 |
| αTRPC | 30 |  | 25,16 | 23,5 | 2,3 | 8284 | 65 |
| αTRPC | 30 | *0.05* | 0,759 |  |  | 8231 | 2 |
| αTRPC | 30 |  | 1,942 | 1,4 | 0,8 | 8282 | 5 |
| αTRPC | 30 | *NTC^d^* | 0 |  |  | 8260 | 0 |
| αTRPC | 30 |  | 0 | 0,0 | 0,0 | 8278 | 0 |
|  |  |  |  |  |  |  |  |
| βTRPC | 24 | *0.2* | 14,47 |  |  | 8280 | 36 |
| βTRPC | 24 |  | 13,14 | 13,8 | 0,9 | 8275 | 35 |
| βTRPC | 24 | *0.15* | 7,778 |  |  | 8256 | 19 |
| βTRPC | 24 |  | 6,763 | 7,3 | 0,7 | 8268 | 17 |
| βTRPC | 24 | *0.1* | 3,662 |  |  | 8230 | 9 |
| βTRPC | 24 |  | 2,788 | 3,2 | 0,6 | 8276 | 7 |
| βTRPC | 24 | *0.05* | 0,8 |  |  | 8258 | 2 |
| βTRPC | 24 |  | 0,389 | 0,6 | 0,3 | 8282 | 1 |
| βTRPC | 24 | *NTC^d^* | 0 |  |  | 8251 | 0 |
| βTRPC | 24 |  | 0,001 | 0,0 | 0,0 | 8275 | 0 |
|  |  |  |  |  |  |  |  |
| βTRPC | 30 | *0.2* | 22,03 |  |  | 8246 | 57 |
| βTRPC | 30 |  | 19,94 | 21,0 | 1,5 | 8271 | 52 |
| βTRPC | 30 | *0.15* | 9,235 |  |  | 8298,0 | 24,0 |
| βTRPC | 30 |  | 10,54 | 9,9 | 0,9 | 8272 | 27 |
| βTRPC | 30 | *0.1* | 5,138 |  |  | 8259 | 13 |
| βTRPC | 30 |  | 3,922 | 4,5 | 0,9 | 8274 | 10 |
| βTRPC | 30 | *0.05* | 0 |  |  | 8306,0 | 0,0 |
| βTRPC | 30 |  | 1,182 | 0,6 | 0,8 | 8264 | 3 |
| βTRPC | 30 | *NTC^d^* | 0 |  |  | 8276 | 0 |
| βTRPC | 30 |  | 0 | 0,0 | 0,0 | 8278 | 0 |
|  |  |  |  |  |  |  |  |
| αTRPM | 24 | *0.2* | 74,95 |  |  | 8254 | 186 |
| αTRPM | 24 |  | 63,75 | 69,35 | 7,9 | 8248 | 166 |
| αTRPM | 24 | *0.15* | 25,61 |  |  | 8306 | 64 |
| αTRPM | 24 |  | 32,45 | 29,03 | 4,8 | 8257 | 85 |
| αTRPM | 24 | *0.1* | 11,4 |  |  | 8286 | 29 |
| αTRPM | 24 |  | 12,85 | 12,125 | 1,0 | 8276 | 34 |
| αTRPM | 24 | *0.05* | 0,391 |  |  | 8242 | 1 |
| αTRPM | 24 |  | 0,753 | 0,572 | 0,2 | 8256 | 2 |
| αTRPM | 24 | *NTC^d^* | 0 |  |  | 8284 | 0 |
| αTRPM | 24 |  | 0,382 | 0,191 | 0,2 | 8254 | 1 |
|  |  |  |  |  |  |  |  |
| αTRPM | 30 | *0.2* | 45,33 |  |  | 8300 | 93 |
| αTRPM | 30 |  | 42,45 | 33,89 | 2,0 | 8257 | 85 |
| αTRPM | 30 | *0.15* | 19,47 |  |  | 8280 | 36 |
| αTRPM | 30 |  | 18,08 | 15,275 | 1,1 | 8277 | 43 |
| αTRPM | 30 | *0.1* | 8,963 |  |  | 8302 | 23 |
| αTRPM | 30 |  | 9,846 | 7,9045 | 1,4 | 8269 | 18 |
| αTRPM | 30 | *0.05* | 0,389 |  |  | 8306 | 1 |
| αTRPM | 30 |  | 0 | 0,1945 | 0,2 | 8253 | 0 |
| αTRPM | 30 | *NTC^d^* | 0 |  |  | 8303 | 0 |
| αTRPM | 30 |  | 0 | 0 | 0 | 8248 | 0 |
|  |  |  |  |  |  |  |  |
| βTRPM | 24 | *0.2* | 14,014 |  |  | 8287 | 20 |
| βTRPM | 24 |  | 13,401 | 13,7 | 0,4 | 8305 | 21 |
| βTRPM | 24 | *0.15* | 6,022 |  |  | 8265 | 12 |
| βTRPM | 24 |  | 5,758 | 5,9 | 0,2 | 8284 | 10 |
| βTRPM | 24 | *0.1* | 2,753 |  |  | 8256 | 2 |
| βTRPM | 24 |  | 2,382 | 2,6 | 0,3 | 8254 | 1 |
| βTRPM | 24 | *0.05* | 0 |  |  | 8259 | 0 |
| βTRPM | 24 |  | 0 | 0,0 | 0,0 | 8248 | 0 |
| βTRPM | 24 | *NTC^d^* | 0 |  |  | 8258 | 0 |
| βTRPM | 24 |  | 0 | 0,0 | 0,0 | 8253 | 0 |
|  |  |  |  |  |  |  |  |
| βTRPM | 30 | *0.2* | 18,963 |  |  | 8302 | 23 |
| βTRPM | 30 |  | 16,07 | 17,5 | 2,0 | 8175 | 26 |
| βTRPM | 30 | *0.15* | 6,245 |  |  | 8267 | 11 |
| βTRPM | 30 |  | 5,855 | 6,1 | 0,3 | 8269 | 10 |
| βTRPM | 30 | *0.1* | 2,758 |  |  | 8281 | 7 |
| βTRPM | 30 |  | 1,965 | 2,4 | 0,6 | 8253 | 5 |
| βTRPM | 30 | *0.05* | 0 |  |  | 8263 | 0 |
| βTRPM | 30 |  | 0 | 0,0 | 0,0 | 8274 | 0 |
| βTRPM | 30 | *NTC^d^* | 0 |  |  | 8251 | 0 |
| βTRPM | 30 |  | 0 | 0,0 | 0,0 | 8247 | 0 |
|  |  |  |  |  |  |  |  |
| αTRPV | 24 | *0.2* | 2,784 |  |  | 8304 | 2 |
| αTRPV | 24 |  | 2,2 | 2,5 | 0,4 | 8272 | 0 |
| αTRPV | 24 | *0.15* | 0 |  |  | 8288 | 0 |
| αTRPV | 24 |  | 0 | 0,0 | 0,0 | 8301 | 0 |
| αTRPV | 24 | *0.1* | 0 |  |  | 8267 | 0 |
| αTRPV | 24 |  | 0 | 0,0 | 0,0 | 8205 | 0 |
| αTRPV | 24 | *0.05* | 0 |  |  | 8250 | 0 |
| αTRPV | 24 |  | 0 | 0,0 | 0,0 | 8094 | 0 |
| αTRPV | 24 | *NTC^d^* |  |  |  |  |  |
| αTRPV | 24 |  |  |  |  |  |  |
|  |  |  |  |  |  |  |  |
| αTRPV | 30 | *0.2* | 15,2 |  |  | 8249 | 54 |
| αTRPV | 30 |  | 17,64 | 16,4 | 1,7 | 8267 | 46 |
| αTRPV | 30 | *0.15* | 6,553 |  |  | 8290 | 17 |
| αTRPV | 30 |  | 7,918 | 7,2 | 1,0 | 8269 | 21 |
| αTRPV | 30 | *0.1* | 1,165 |  |  | 8292 | 3 |
| αTRPV | 30 |  | 3,455 | 2,3 | 1,6 | 8158 | 9 |
| αTRPV | 30 | *0.05* | 0,776 |  |  | 8295 | 2 |
| αTRPV | 30 |  | 0,38 | 0,6 | 0,3 | 8267 | 1 |
| αTRPV | 30 | *NTC^d^* | 0 |  |  | 8289 | 0 |
| αTRPV | 30 |  | 0 | 0,0 | 0,0 |  |  |
|  |  |  |  |  |  |  |  |
| TRPP | 24 | *0.2* | 35 |  |  | 8293 | 96 |
| TRPP | 24 |  | 29,76 | 32,4 | 3,7 | 8257 | 85 |
| TRPP | 24 | *0.15* | 14,869 |  |  | 8280 | 36 |
| TRPP | 24 |  | 15,542 | 15,2 | 0,5 | 8280 | 36 |
| TRPP | 24 | *0.1* | 6,814 |  |  | 8269 | 15 |
| TRPP | 24 |  | 5,526 | 6,2 | 0,9 | 8259 | 13 |
| TRPP | 24 | *0.05* | 0,378 |  |  | 8273 | 1 |
| TRPP | 24 |  | 0 | 0,2 | 0,3 | 8251 | 0 |
| TRPP | 24 | *NTC^d^* | 0 |  |  | 8253 | 0 |
| TRPP | 24 |  | 0 |  |  | 8278 | 0 |
|  |  |  |  |  |  |  |  |
| TRPP | 30 | *0.2* | 48 |  |  | 8248 | 166 |
| TRPP | 30 |  | 53,76 | 50,9 | 4,1 | 8255 | 175 |
| TRPP | 30 | *0.15* | 14,869 |  |  | 8305 | 39 |
| TRPP | 30 |  | 15,542 | 15,2 | 0,5 | 8277 | 43 |
| TRPP | 30 | *0.1* | 6,814 |  |  | 8269 | 15 |
| TRPP | 30 |  | 5,526 | 6,2 | 0,9 | 8259 | 13 |
| TRPP | 30 | *0.05* | 0,378 |  |  | 8249 | 1 |
| TRPP | 30 |  | 0 | 0,2 | 0,3 | 8307 | 0 |
| TRPP | 30 | *NTC^d^* | 0 |  |  | 8230 | 0 |
| TRPP | 30 |  | 0 | 0,0 | 0,0 | 8276 | 0 |
|  |  |  |  |  |  |  |  |
| TRPML | 24 | *0.2* | 3,169 |  |  | 8301 | 8 |
| TRPML | 24 |  | 2,756 | 3,0 | 0,3 | 8289 | 7 |
| TRPML | 24 | *0.15* | 1,192 |  |  | 8272 | 3 |
| TRPML | 24 |  | 1,616 | 1,4 | 0,3 | 8267 | 4 |
| TRPML | 24 | *0.1* | 0,398 |  |  | 8280 | 1 |
| TRPML | 24 |  | 0,38 | 0,4 | 0,0 | 8267 | 1 |
| TRPML | 24 | *0.05* | 0 |  |  | 8279 | 0 |
| TRPML | 24 |  | 0 | 0,0 | 0,0 | 8264 | 0 |
| TRPML | 24 | *NTC^d^* | 0 |  |  | 8291 | 0 |
| TRPML | 24 |  | 0 | 0,0 | 0,0 | 8274 | 0 |
|  |  |  |  |  |  |  |  |
| TRPML | 30 | *0.2* | 16,64 |  |  | 8265 | 44 |
| TRPML | 30 |  | 13,4 | 15,0 | 2,3 | 8290 | 36 |
| TRPML | 30 | *0.15* | 7,335 |  |  | 8269 | 19 |
| TRPML | 30 |  | 7,791 | 7,6 | 0,3 | 8260 | 20 |
| TRPML | 30 | *0.1* | 1,97 |  |  | 8260 | 5 |
| TRPML | 30 |  | 3,138 | 2,6 | 0,8 | 8284 | 8 |
| TRPML | 30 | *0.05* | 0 |  |  | 8292 | 0 |
| TRPML | 30 |  | 0 | 0,0 | 0,0 | 8289 | 0 |
| TRPML | 30 | *NTC^d^* | 0 |  |  | 8273 | 0 |
| TRPML | 30 |  | 0 | 0,0 | 0,0 | 8289 | 0 |

**^a^** Number of target gene (copies/µL) in whole embryos dilution series measured by dPCR and estimated by a Poisson distribution to the ratio of positive to negative partitions.

**^b^** Total number of partitions in dPCR reaction.

**^c^** Number of partitions showing a positive signal for each target gene.

**^d^** NTC= no template control.

**Supplemental Figure S1. Serially diluted cDNA**

**
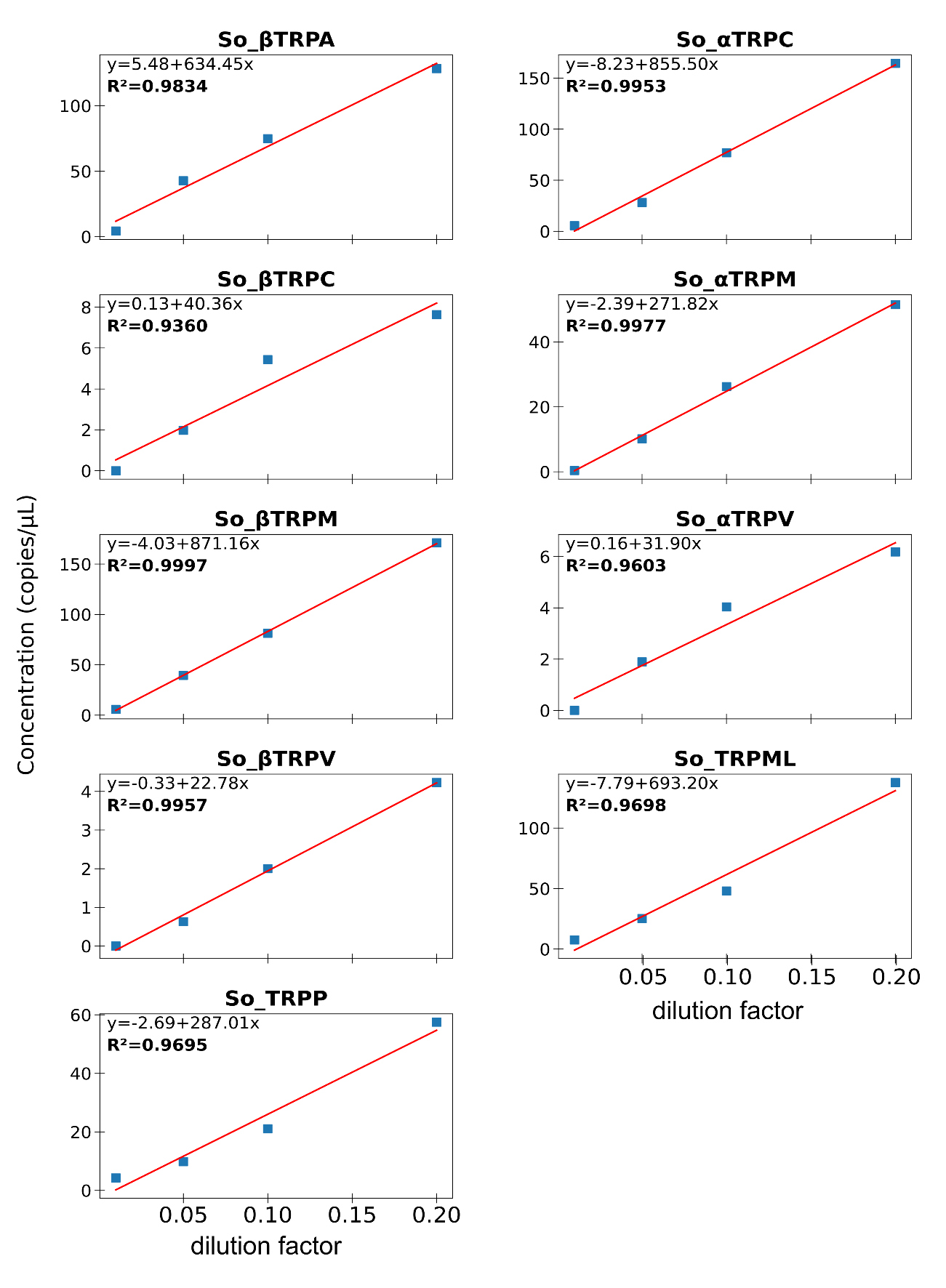
**

**Figure S1**. Estimated copy number/µL concentration by dPCR for serially diluted cDNA analysed in tandem from target primers in *Sepia officinalis*.

**Supplemental Figure S2. Serially diluted cDNA**


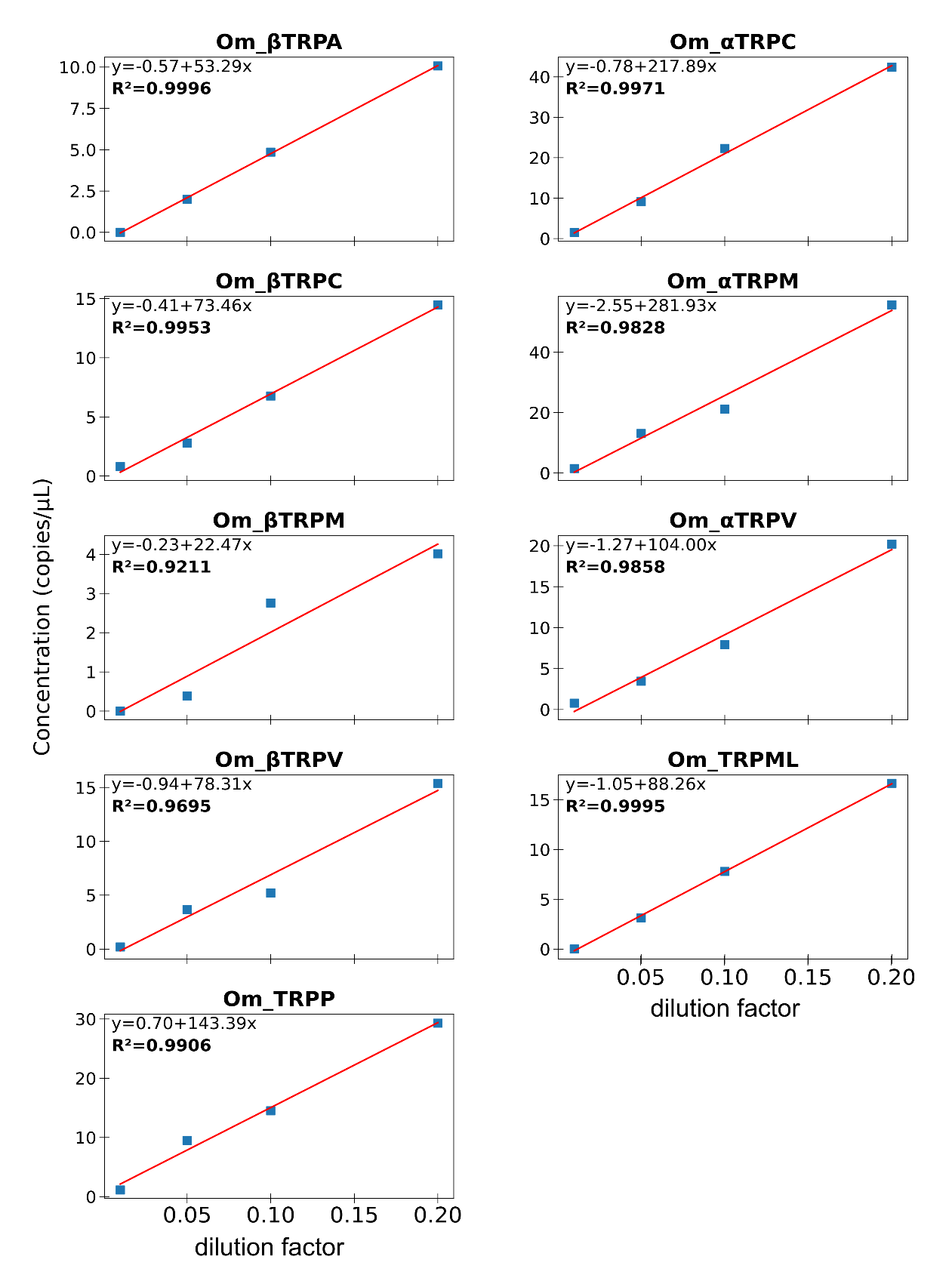


**Figure S2**. Estimated copy number/µL concentration by dPCR for serially diluted cDNA analysed in tandem from target primers in *Octopus maya*.

**Supplemental Figure S3.** Representative visualizations of amplification results obtained by dPCR using serially diluted cDNA with target-specific primers.


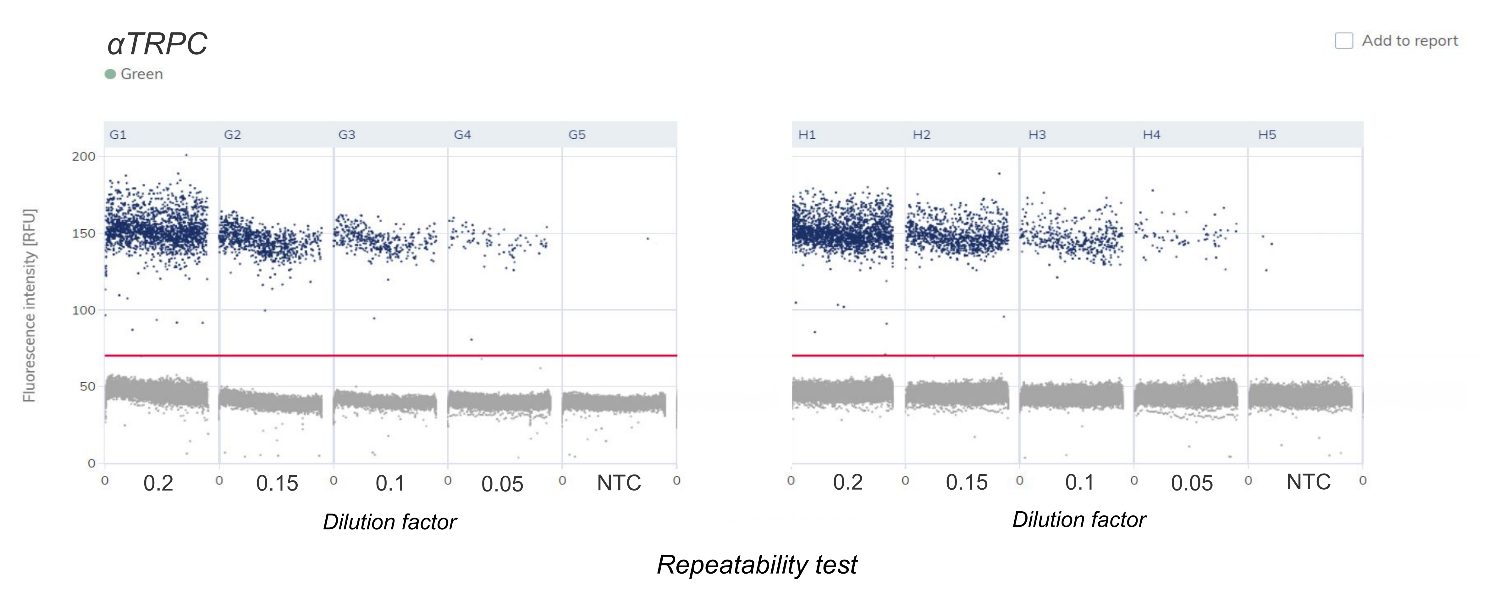


**Figure S3**. Representative example of target gene amplification across serial dilutions using digital PCR (dPCR). The EvaGreen fluorescence signal (y-axis) is plotted against individual partitions (x-axis). NTC: No Template Control (no significant amplification was observed).

**Supplemental Figure S4.**

**

**

**Figure S4. Global phylogenetic relationships of metazoan TRP channel sequences.** The tree summarizes the phylogenetic relationships inferred from an alignment of 334 TRP amino acid sequences, including 250 non-vertebrate sequences—among them representatives from multiple cephalopod species—and 84 vertebrate sequences. Phylogenetic relationships were reconstructed using Maximum Likelihood (RAxML) and Bayesian Inference (MrBayes) analyses. Node values are shown as Maximum Likelihood bootstrap support percentages and Bayesian posterior probabilities (BS/PP). The piezo sequence from *Drosophila melanogaster* (NM_001273298.1) was used as the outgroup to root the tree. Seven major TRP channel families (TRPA, TRPN, TRPV, TRPC, TRPML, TRPP, and TRPM) are indicated. *Sepia officinalis* and *Octopus maya* sequences are highlighted in bold.

**Supplemental Figure S5.**

**
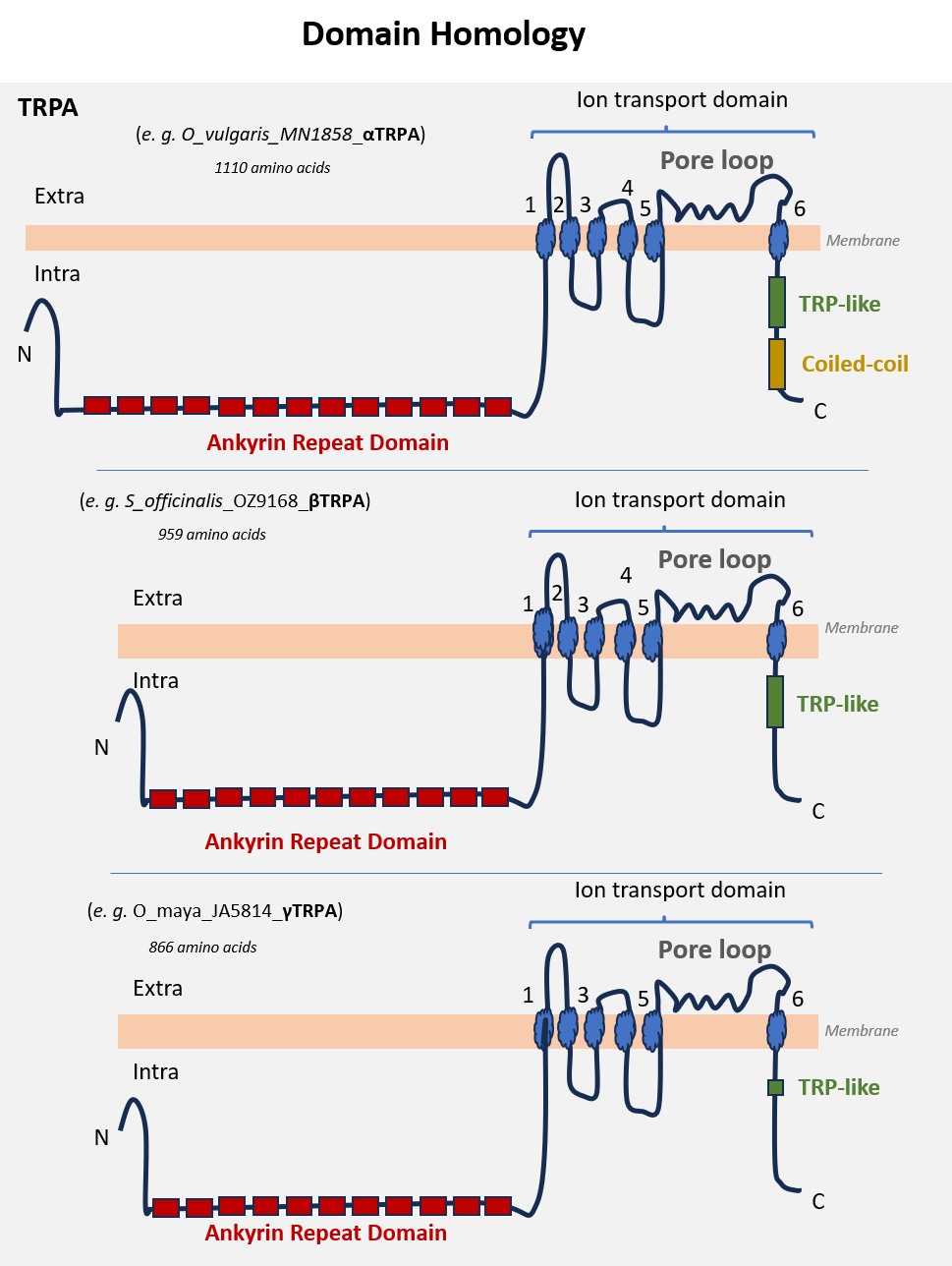
**

**Figure S5. Predicted domain architecture of representative cephalopod TRPA channels.** Three representative TRPA subtype are shown: αTRPA from *Octopus vulgaris* (MN1858), βTRPA from *Sepia officinalis* (OZ9168), and γTRPA from *Octopus maya* (JA5814). All proteins display the characteristic TRPA architecture, including N-terminal ankyrin repeat domains, a six-transmembrane ion transport domain (TM1–TM6) with a pore-forming region between TM5 and TM6, and a C-terminal TRP-like domain. Despite variation in protein length (1110, 959, and 866 amino acids, respectively), the major structural features are conserved among the three cephalopod TRPA subtypes. Schematics were adapted from previously published TRPA channel representations (Himmel et al., 2020; Himmel and Cox, 2020) and revised using representative cephalopod TRPA sequences identified in the present study, with domain architecture inferred from InterProScan, NCBI CDD, and Protter analyses.

**Supplemental Figure S6.**

**
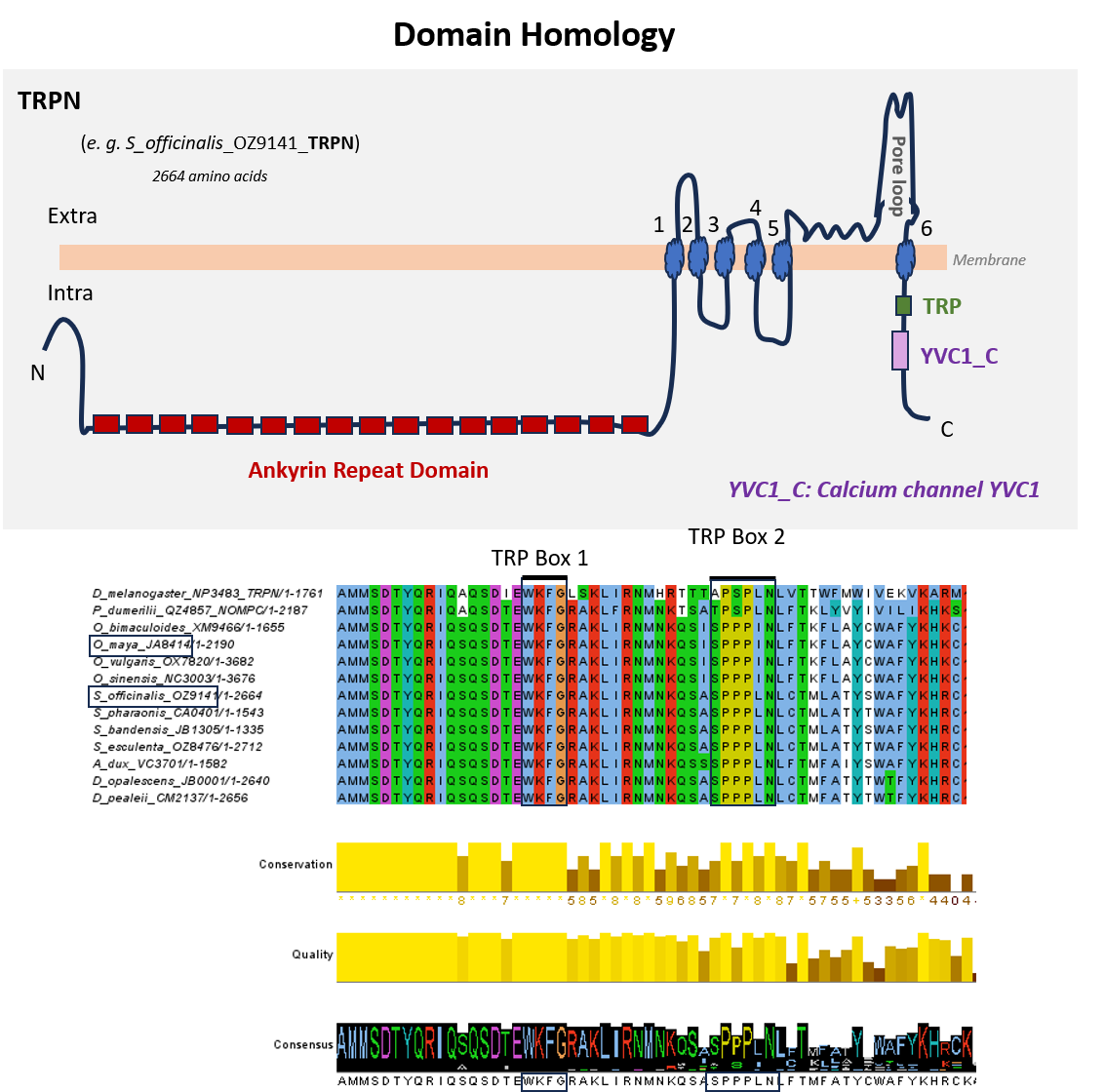
**

**Figure S6. Predicted domain architecture of a representative cephalopod TRPN channel and conservation of the TRP domain.** A representative TRPN homologue from *Sepia officinalis* (OZ9141) is shown. The protein contains multiple N-terminal ankyrin repeat domains, a six-transmembrane ion transport domain (TM1–TM6) with a pore-forming region between TM5 and TM6, and a C-terminal YVC1_C domain. The predicted protein comprises 2664 amino acids, consistent with the large size typically observed in TRPN/NOMPC channels. The lower panels show the multiple sequence alignment of the TRP domain from representative cephalopod TRPN homologues, together with conservation scores and the derived consensus sequence. The highly conserved TRP-box motif is highlighted, revealing sequence conservation across cephalopod TRPN channels. Schematics were adapted from previously published TRPN channel representations (Himmel et al., 2020; Himmel and Cox, 2020) and revised using representative cephalopod TRPN sequences identified in the present study, with domain architectures inferred from InterProScan, NCBI CDD, and Protter analyses.

**Supplemental Figure S7.**

**
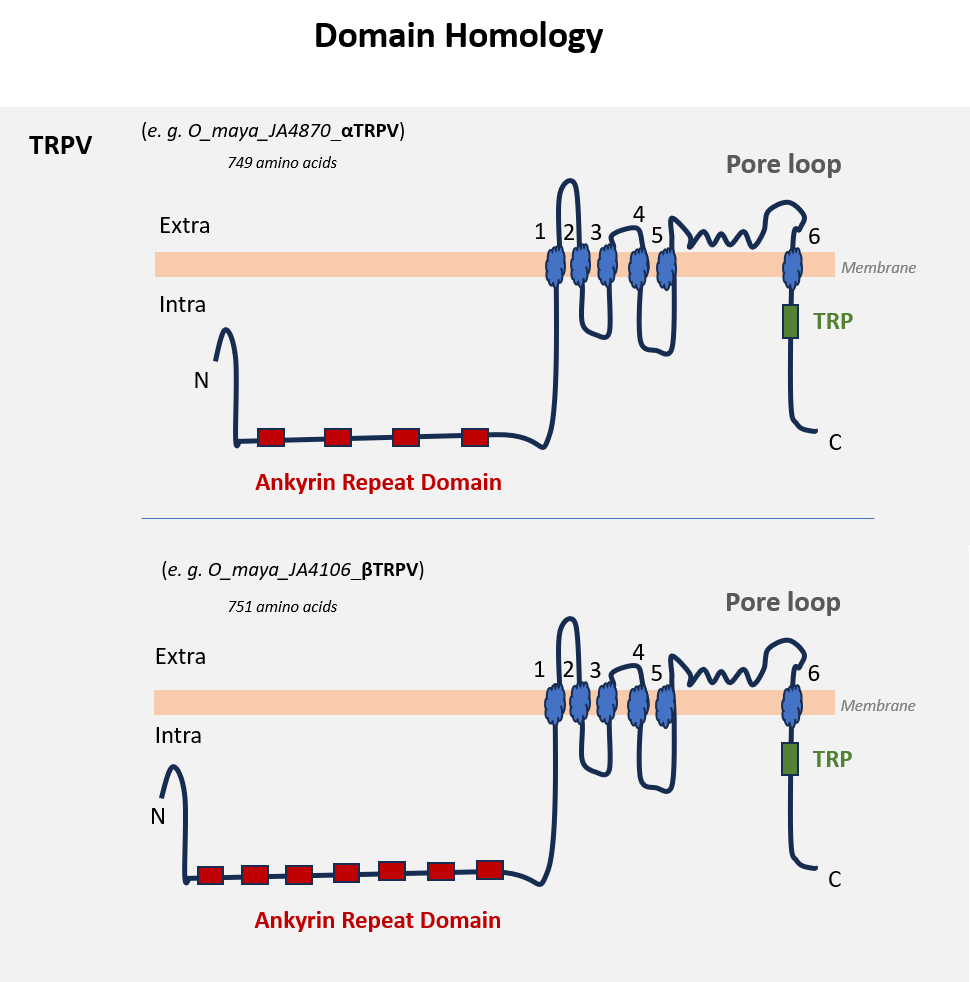
**

**Figure S7. Predicted domain architecture of representative cephalopod TRPV channels.** Two representative TRPV subtypes are shown: αTRPV from *O. maya* (JA4870) and βTRPV from *O. maya* (JA4106). Both proteins contain N-terminal ankyrin repeat domains, a six-transmembrane ion transport domain (TM1–TM6) with a pore-forming region between TM5 and TM6, and a C-terminal TRP domain. The representative αTRPV and βTRPV proteins comprise 749 and 751 amino acids, respectively. Variation in the number of predicted N-terminal ankyrin repeats is observed between the two proteins, whereas the overall domain architecture remains similar. Schematics were adapted from previously published TRPV channel representations (Himmel et al., 2020; Himmel and Cox, 2020) and revised using representative cephalopod TRPV sequences identified in the present study, with domain architectures inferred from InterProScan, NCBI CDD, and Protter analyses.

**Supplemental Figure S8.**

**
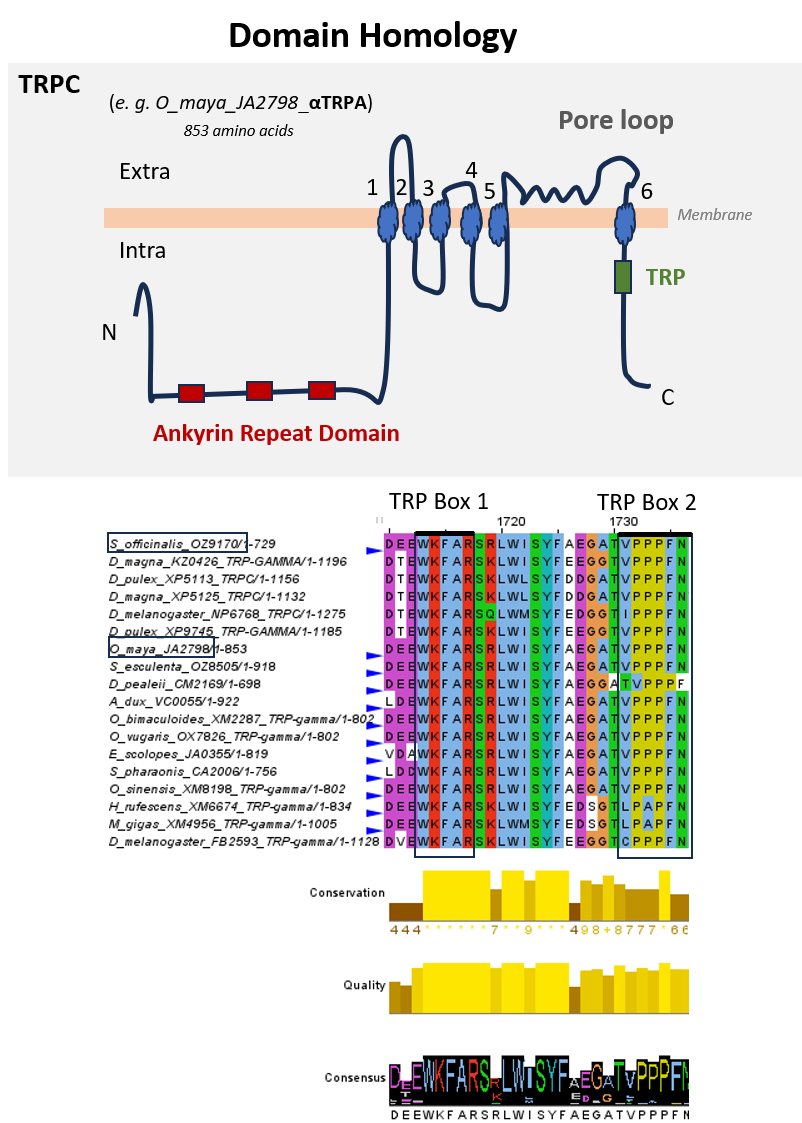
**

**Figure S8. Predicted domain architecture of a representative cephalopod TRPC channel and conservation of the TRP domain.** A representative αTRPC homologue from *Octopus maya* (JA2798) is shown. The predicted protein contains N-terminal ankyrin repeat domains, a six-transmembrane ion transport domain (TM1–TM6) with a pore-forming region between TM5 and TM6, and a C-terminal TRP domain. The representative protein comprises 853 amino acids, which falls within the size range commonly reported for TRPC channels. The lower panels show the multiple sequence alignment of the TRP domain from representative TRPC homologues, together with conservation scores and the corresponding consensus sequence. The TRP-box motif is indicated and represents one of the most conserved regions among the sequences analysed. Schematics were adapted from previously published TRPV channel representations (Himmel et al., 2020; Himmel and Cox, 2020) and revised using representative cephalopod TRPC sequences identified in the present study, with domain architectures inferred from InterProScan, NCBI CDD, and Protter analyses.

**Supplemental Figure S9.**

**
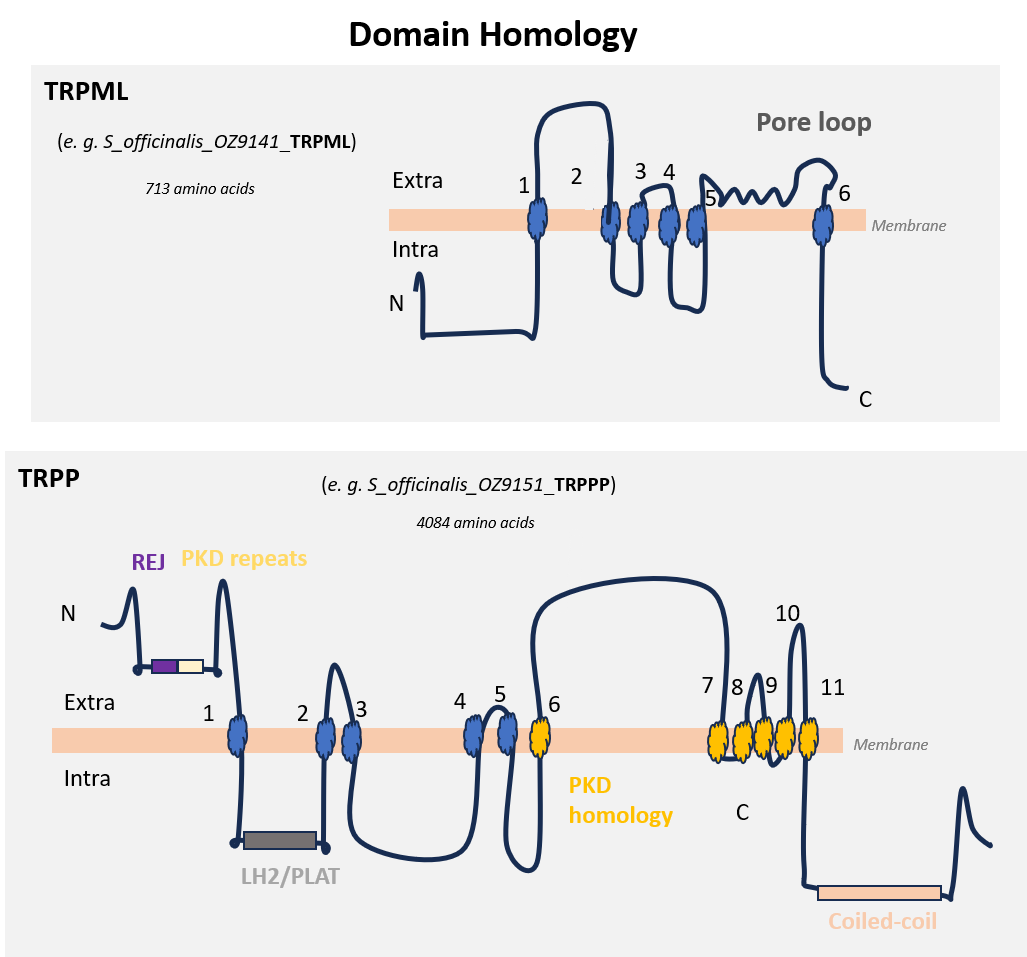
**

**Figure S9. Predicted domain architectures of representative cephalopod TRPML and TRPP channels.** Representative TRPML (*Sepia officinalis*, OZ9141) and TRPP (*S. officinalis*, OZ9151) homologues are shown. The TRPML protein contains a six-transmembrane ion transport region with a pore-forming loop between TM5 and TM6, whereas the TRPP protein exhibits a more complex architecture comprising REJ (receptor for egg jelly) and PKD repeat domains, an LH2/PLAT (Lipoxygenase homology/ Polycystin-1, Lipoxygenase, Alpha-Toxin) domain, a PKD homology region, an eleven-transmembrane segment, and a C-terminal coiled-coil domain. Schematics were adapted from previously published TRPML and TRPP channels representations (Himmel et al., 2020; Himmel and Cox, 2020) and revised using representative cephalopod sequences identified in the present study, with domain architectures inferred from InterProScan, NCBI CDD, and Protter analyses.

**Supplemental Figure S10.**

**
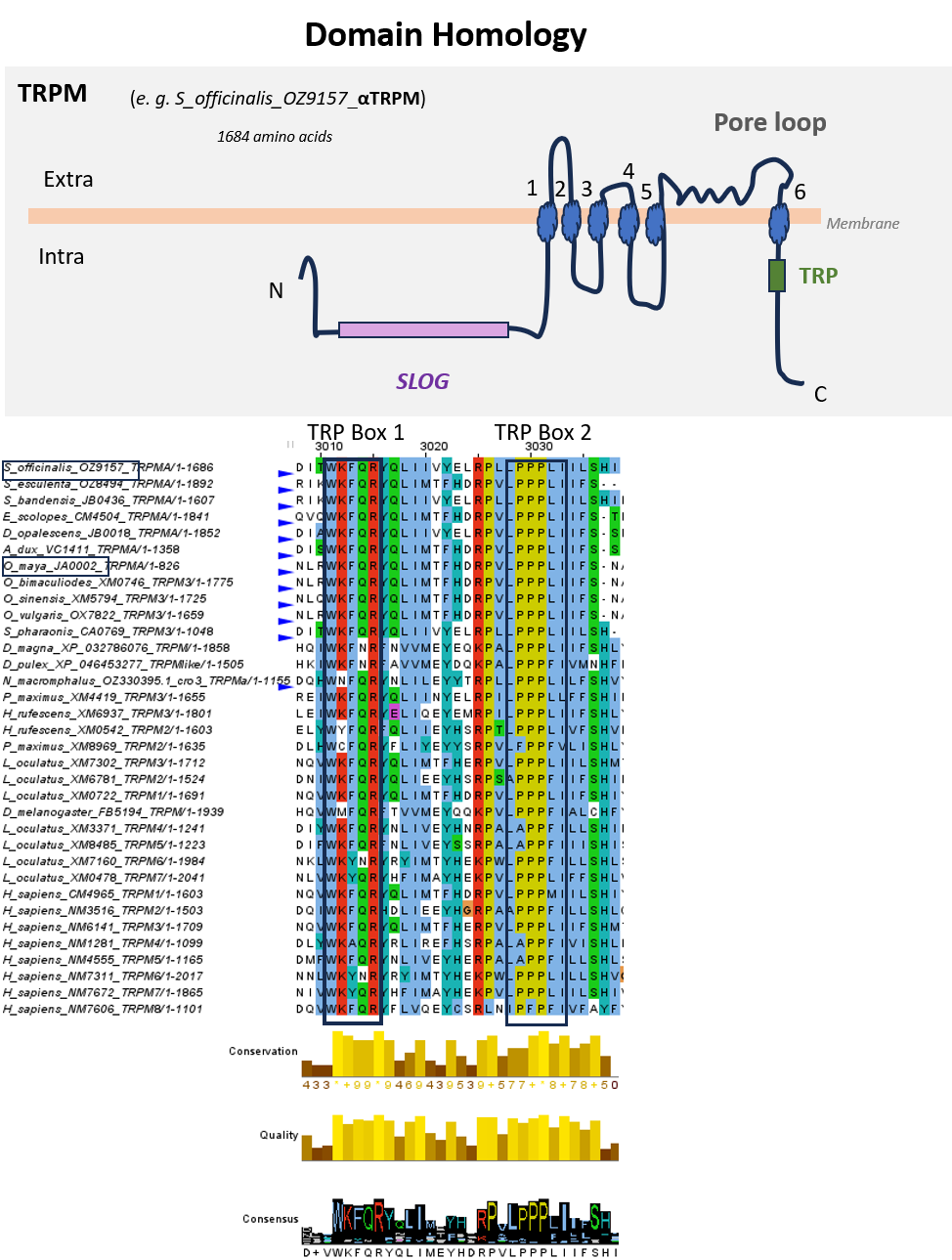
**

**Figure S10. Predicted domain architecture of a representative cephalopod TRPM channel and conservation of the TRP domain.** A representative αTRPM homologue from *Sepia officinalis* (OZ9157) is shown. The predicted protein comprises 1684 amino acids and contains an N-terminal SLOG domain, a six-transmembrane ion transport domain (TM1–TM6) with a pore-forming region between TM5 and TM6, and a C-terminal TRP domain. The lower panels show a multiple sequence alignment of the TRP domain from representative TRPM homologues, together with conservation scores and the derived consensus sequence. The TRP-box motif is highlighted and corresponds to one of the most conserved regions within the alignment. Schematics were adapted from previously published TRPM channel representations (Himmel et al., 2020; Himmel and Cox, 2020) and revised using representative cephalopod TRPM sequences identified in the present study, with domain architectures inferred from InterProScan, NCBI CDD, and Protter analyses.

**Supplemental Figure S11.**

**
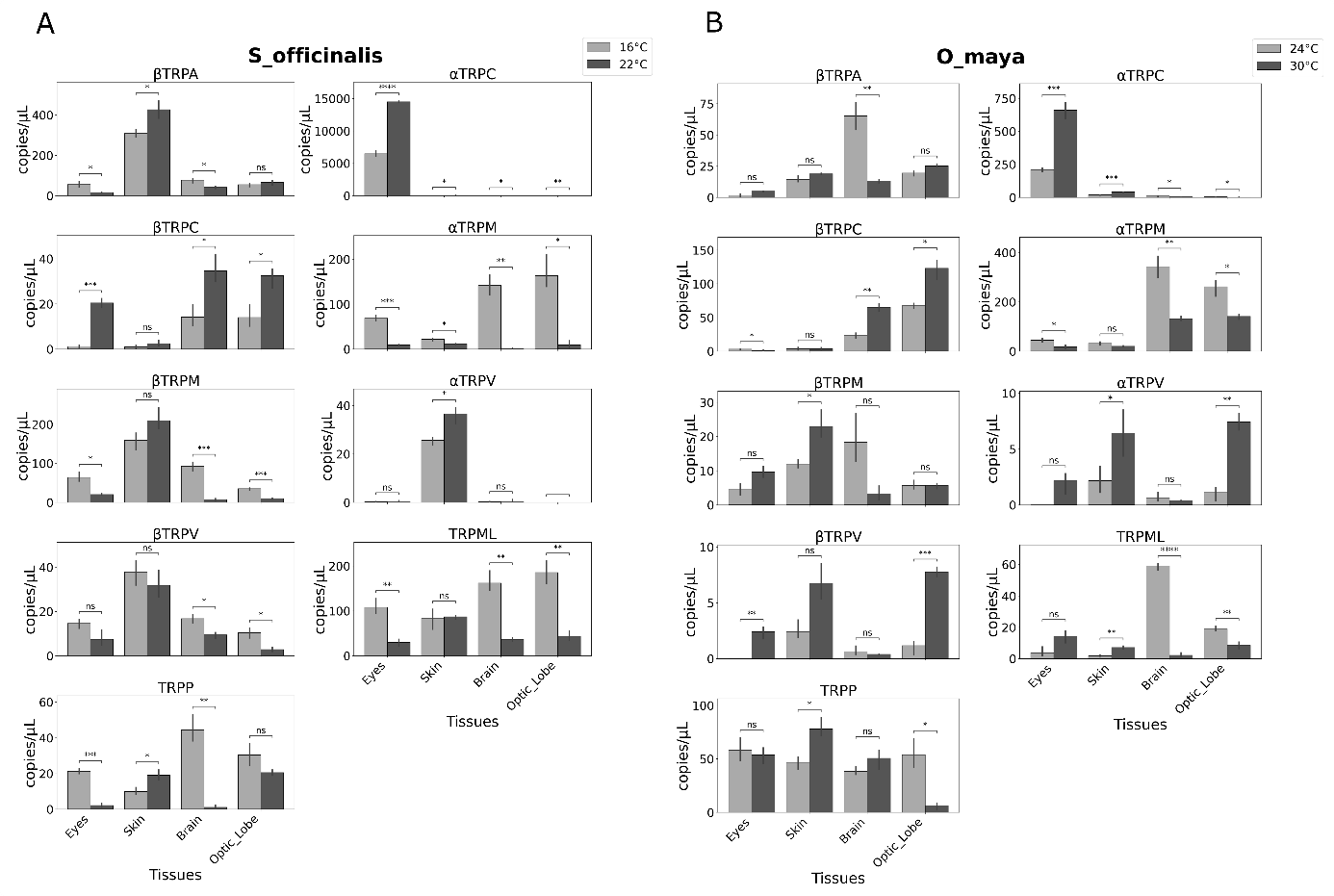
**

**Figure S5**. Expression profile of TRP genes (copies/µL, absolute quantification dPCR) across four distinct tissues (eyes, skin, optic lobes, and brain) in *S. officinalis and O. maya* embryos exposed to heat temperature stress. (A) Gene expression profile in *S. officinalis* after 20 days of chronic exposure to optimal (16°C) and critically high (22°C) temperatures. (B) Gene expression profile in *O. maya* after 30 days of chronic exposure to optimal (24°C) and heat stress (30°C) temperatures. Statistical significance was assessed by Student’s t-test (*n*=4; *p <0.05, **p <0.01, ***p <0.001, ****p <0.0001, and ns = no significative).

**References**

Himmel, N.J., Cox, D.N., 2020. Transient receptor potential channels: current perspectives on evolution, structure, function and nomenclature. Proc. R. Soc. B Biol. Sci. 287, 20201309. https://doi.org/10.1098/rspb.2020.1309

Himmel, N.J., Gray, T.R., Cox, D.N., 2020. Phylogenetics Identifies Two Eumetazoan TRPM Clades and an Eighth TRP Family, TRP Soromelastatin (TRPS). Mol. Biol. Evol. 37, 2034–2044. https://doi.org/10.1093/molbev/msaa065
